# Plague Transmission from Corpses and Carcasses

**DOI:** 10.3201/eid2708.200136

**Published:** 2021-08

**Authors:** Sophie Jullien, Nipun Lakshitha de Silva, Paul Garner

**Affiliations:** University of Barcelona, Barcelona, Spain (S. Jullien);; Liverpool School of Tropical Medicine, Liverpool, UK (S. Jullien, P. Garner);; General Sir John Kotelawala Defence University, Colombo, Sri Lanka (N.L. de Silva)

**Keywords:** plague, *Yersinia pestis*, outbreaks, corpses, body fluids, *Y. pestis*, bacterial infections, bacteria, vector-borne infections, zoonoses, bacterial zoonoses, carcasses

## Abstract

Knowing whether human corpses can transmit plague will inform policies for handling the bodies of those who have died of the disease. We analyzed the literature to evaluate risk for transmission of *Yersinia pestis*, the causative agent of plague, from human corpses and animal carcasses. Because we could not find direct evidence of transmission, we described a transmission pathway and assessed the potential for transmission at each step. We examined 3 potential sources of infection: body fluids of living plague patients, infected corpses and carcasses, and body fluids of infected corpses. We concluded that pneumonic plague can be transmitted by intensive handling of the corpse or carcass, presumably through the inhalation of respiratory droplets, and that bubonic plague can be transmitted by blood-to-blood contact with the body fluids of a corpse or carcass. These findings should inform precautions taken by those handling the bodies of persons or animals that died of plague.

Plague is an ancient disease that has killed millions of persons including one third of the population of Europe during the Black Death pandemic in the 14th century (*1*). Plague remains a threat in many parts of the world (*2*) and has been categorized by the World Health Organization as a reemerging disease (*3*). Caused by *Yersinia pestis*, a nonmotile, gram-negative coccobacillus, this zoonotic disease has its main reservoir in rodents (*4*,*5*). Humans become infected by *Y. pestis* through bites from infected fleas or animals, handling or ingesting infected animals or humans, or inhaling aerosolized droplets from infected tissues (Figure 1) (*6*–*10*). Plague has 3 main clinical syndromes: bubonic plague, which is characterized by inflammation of lymph nodes after a flea bite or scratch from an infected animal (*11*,*12*); pneumonic plague, which is spread by inhalation of droplets from infected humans or animals; and septicemic plague, which results from the hematogenous spread of bubonic or pneumonic plague (*13*).

**Figure 1:**
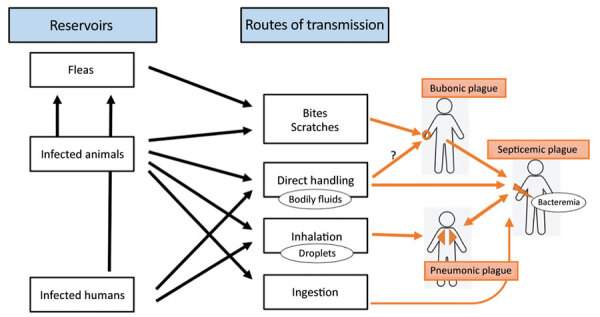
Reservoirs of *Yersinia pestis* and transmission routes leading to different forms of plague. Black arrows indicate links between infection sources and transmission routes. Orange arrows indicate causality of different plague syndromes according to transmission routes.

To inform World Health Organization recommendations on personal protective equipment (PPE) for healthcare workers, we evaluated whether corpses of plague patients might be infectious. Little is known about the potential infectiousness of corpses, the duration of risk for infection to humans handling corpses, or possible transmission routes. Information on infectiousness of human corpses can guide development of protective measures for healthcare staff and relatives who might not use PPE during traditional funeral rituals (*14*). We know of 3 possible transmission routes: direct contact with infectious body fluids, such as through open wounds or inhalation; indirect contact through contaminated clothing; and bites from infected fleas from corpses or their clothes. In this review, we sought to estimate the risk for *Y. pestis* transmission from body fluids of corpses. Because little direct evidence for plague transmission from corpses exists, we assessed evidence for potential transmission by body fluids of living plague patients, corpses and carcasses, and body fluids of corpses and carcasses. We also analyzed the potential duration of infectiousness of body fluids from corpses and carcasses (Figure 2) (*15*).

**Figure 2:**
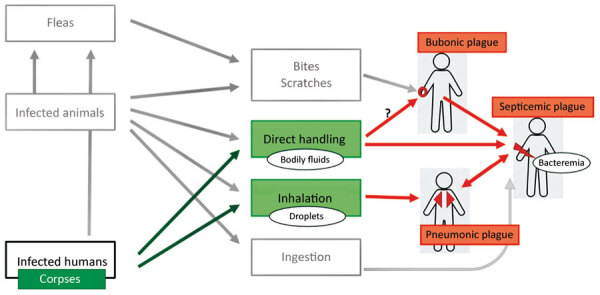
Potential plague transmission routes from human corpses. Black arrows indicate links between infection sources and transmission routes. Orange arrows indicate causality of different plague syndromes according to transmission routes.

## Methods

We used different inclusion criteria for each potential transmission pathway (Table 1). Because we assumed that the consumption of human corpses was rare, we excluded cases caused by the consumption of infected meat. We also excluded cases caused by transmission from vectors, such as fleas.

**Table 1:** Inclusion criteria for literature review on transmission of plague from human corpses

Research topic	Infectiousness of body fluids of living plague patients	Infections acquired from corpses and carcasses	Infectiousness of body fluids of corpses and carcasses
Study type	Descriptive (including surveillance data, case series, and case reports)	Descriptive (including case series and case reports)	Descriptive (including case series and case reports)
Participants	Persons who have laboratory-confirmed plague	Persons or animals that died of laboratory-confirmed plague	Persons or animals that died of laboratory-confirmed plague
Outcomes	New case of confirmed plague attributed to direct transmission from an infected human (i.e., human-to-human transmission)	New case of confirmed plague attributed to direct transmission from an infected corpse or carcass	New case of confirmed plague attributed to direct transmission from an infected corpse or carcass, with a specified period between the time of death of the plague victim and time of contact with corpse
			Isolation of *Yersinia pestis* by culture from body fluids from an infected corpse or carcass, with a specified period between the time of death of the plague victim and the time of *Y. pestis* identification
Exclusion criteria	None	Studies reporting only cases of plague attributed to consumption of infected meat, or cases transmitted by vectors such as fleas	Studies examining the persistence of *Y. pestis* DNA in corpses or carcasses that were previously buried, in the soil, or on environmental surfaces

We searched PubMed, Embase, Science Citation Index, and Scopus for literature published by May 20, 2019, and identified all relevant studies regardless of language, publication status, or publication date (Appendix). We also manually searched the reference lists of all identified papers and contacted relevant researchers.

### Study Selection

**Figure 3:**
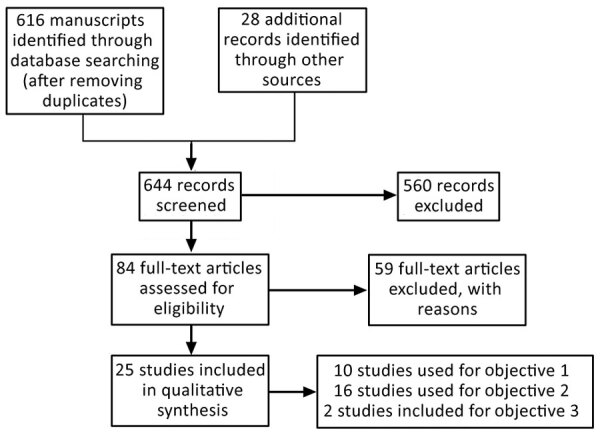
Flowchart of study on plague transmission from human corpses. Study examined 3 potential sources of infection: body fluids of living plague patients (objective 1), infected corpses and carcasses (objective 2), and body fluids of infected corpses (objective 3).

First, we (2 review authors) independently screened the abstracts of articles retrieved by the search strategy and classified them using predefined eligibility criteria (Table 1). For the second stage of screening, we retrieved full-text copies and applied the same criteria. We assessed manuscripts in French, Russian, German, and Chinese with the help of native-speaking authors and plague experts or through online translation. We resolved any discrepancies through discussion and excluded studies that did not meet the inclusion criteria (Figure 3; Appendix Table 1).

### Data Extraction, Bias Assessment, and Analysis

For each included study, we (2 review authors) extracted data on protocol and other characteristics (Appendix Tables 4–57). We also considered each study’s limitations by assessing risk for bias using 6 questions modified from the quality appraisal tool developed by Cho et al. (*16*) (Appendix Table 3). We did not find suitable data for statistical analysis.

## Results

We identified 644 studies (616 in the literature search, after removal of duplicates, and 28 in the manual search) and used 25 in the final review (Figure 3). Ten studies addressed potential transmission by body fluids of living persons who had plague, 16 addressed potential transmission from corpses and carcasses, and 2 addressed potential transmission from body fluids of human corpses and animal carcasses. Three studies addressed >1 research question.

### Infectiousness of Body Fluids of Living Plague Patients

#### Study Descriptions

We found 10 studies that documented direct human-to-human transmission of *Y. pestis* (Appendix Table 4). In total, 4 studies described plague cases during the 20th century in Brazil (*17*), South Africa (*18*), and the United States (*19*,*20*) and 6 reported outbreaks during 1997–2017 in Madagascar (*21*–*24*), Uganda (*25*), and the Democratic Republic of the Congo (*26*). Altogether, the 10 studies described 2,388 plague cases caused by direct human-to-human contact, including 1,861 cases documented during an outbreak in Madagascar (*21*). Nearly all the patients had primary pneumonic plague, except for 4 patients who had septicemic plague (*18*,*26*) and 6 who had a mixed form described as probable pneumonic affectation secondary to buboes (*18*).

#### Risk for Bias

Six studies included adequate descriptions of patient characteristics such as age, sex, and form of plague; 3 had inadequate descriptions; and 1 did not provide such information. Four studies described efforts to trace contacts from the index case, suggesting a perception of contagiousness. All 10 studies met our inclusion criterion by providing a description of laboratory methods used to confirm cases, although 2 studies included only partial descriptions. We used the quality appraisal tool to judge whether the suggested transmission route and causative relationship to infection was plausible for 8 studies. We could not make definitive judgements for 2 studies that comprised 50 cases but lacked sufficient data (Table 2; Appendix Tables 6, 8, 10, 12, 14, 16, 18, 20, 22, 24).

**Table 2:** Risk for bias in studies on human-to-human transmission of plague*

Study	Were patient characteristics adequately reported?	Was there some effort to trace all contacts from the index case?	Were the methods used for tracing contacts adequate?	Were the laboratory methods used for defining a confirmed case of plague reliable?	Was the route of transmission plausible?	Was the cause-effect of transmission plausible?
Almeida et al. (*17*)	Partial	Unknown	NA	Yes	No	Unknown
Begier et al. (*25*)	Yes	Yes	Yes	Partial	Yes	Yes
Bertherat et al. (*26*)	No	Partial	Unknown	Yes	Yes	Yes
Evans et al. (*18*)	Yes	Unknown	NA	Yes	Yes	Yes
Kellogg et al. (*20*)	Yes	Unknown	NA	Yes	Yes	Yes
Kugeler 2015 (*19*)	Partial	Unknown	NA	Yes	Unknown	Unknown
Rabaan et al. (*21*)	Partial	Partial	Unknown	Yes	Yes	Yes
Ramasindrazana et al. (*22*)	Yes	Yes	Yes	Yes	Yes	Yes
Ratsitorahina et al. (*23*)	Yes	Yes	Unknown	Partial	Yes	Yes
Richard et al. (*24*)	Yes	Yes	Unknown	Yes	Yes	Yes

*NA, not applicable.

#### Findings

Various studies reported bloody sputum from the index patient (*23*,*25*), infected contacts (*18*,*22*), or both (*24*). Transmission was attributed to respiratory droplets for 1,893 combined cases (*20*,*21*,*23*,*25*) and to aerosolized bacteria for 311 combined cases (*24*,*26*). A combination of 3 studies found that 63 cases were consistent with human-to-human transmission, but the studies did not provide further details (*17*,*19*,*22*).

To assess the contagiousness of plague patients, we extracted data about uninfected contacts. Across 4 studies that provided such information, a total of 51 contacts were infected by 5 index patients (although however, some infected contacts then acted as index patients for additional infections), whereas 341 contacts of those 5 index patients did not become ill (*22*–*25*). The study authors estimated incidence proportions of 8%, 8.4%, and 55% (*23*–*25*). One study estimated the transmission rate to be 0.41 susceptible persons/day (*22*). Some studies reported that infected contacts had close and prolonged exposure to index patients (*18*,*20*,*23*–*25*). Four studies from South Africa and Madagascar attributed plague transmission to funerary activities, such as preparing bodies for funerals or active participation in the funerals (*18*,*21*–*23*). Uninfected contacts included family members who slept in the same bed as the patient until the night before the patient’s death (*24*,*25*); some of these contacts slept with their heads <2 meters from the coughing plague patient (*25*).

#### Summary

In total, 6 studies described 2,204 cases of direct *Y. pestis* transmission through infective cough droplets from living plague patients. Some direct transmission occurred only after close and prolonged exposure. We found no publication describing human-to-human transmission of plague through other body fluids, such as blood (although patients with pneumonic plague can produce respiratory droplets from bloody sputum), urine, feces, sweat, or bubo pus.

### Plague Transmitted by Corpses and Carcasses

#### Study Descriptions

We analyzed 16 retrospective case reports and series published during 1930–2019 (Appendix Tables 25–57). The studies documented a total of 250 cases in 7 countries: 114 in China (*27*–*29*), 96 in the United States (*8*,*19*,*30*–*35*), 17 in Libya (*36*), 12 in Kazakhstan (*37*), 9 in Madagascar (*23*), 1 in South Africa (*38*), and 1 in Saudi Arabia (*39*). Plague was more common among men than women, and patient ages ranged from 1–69 years. The combined studies reported 125 cases of primary bubonic plague (mostly with axillary buboes), 70 of primary pneumonic plague, 8 of primary septicemic plague, and 2 of primary intestinal plague.

#### Risk for Bias

Ten studies adequately described the main characteristics of participants (Table 3; Appendix Tables 27, 29, 31, 33, 35, 37, 39, 41, 43, 45, 47, 49, 51, 53, 55, 57). Twelve studies did not describe efforts to trace all contacts of the index patient. These studies provided no information on whether other persons were exposed but did not get infected, complicating our assessment of corpse contagiousness. Eight studies had missing or partial descriptions of laboratory methods used for defining confirmed cases of plague; however, patients with unconfirmed infection were highly suspected to have plague because of clinical and epidemiologic data. Using the quality appraisal toll, we judged the proposed transmission route and causative relationship to infection to be highly plausible in 11 studies. Although the remaining 5 studies and case series described in an additional 2 sources also proposed transmission routes, they lacked the information needed to judge plausibility. Furthermore, some case series could not fully exclude fleaborne transmission in all patients.

**Table 3:** Risk for bias summary in studies on plague acquired from corpses and carcasses

Study ID	Were patient characteristics adequately reported?	Was there some effort to trace all contacts from the index case?	Were the methods used for tracing contacts adequate?	Were the laboratory methods used for defining a confirmed case of plague reliable?	Was the route of transmission plausible?	Was the cause-effect of transmission plausible?
Centers for Disease Control and Prevention (*30*)	Yes	Unknown	NA	Yes	Yes	Yes
Christie et al. (case series 1; *37*)	Partial	Unknown	NA	Partial	Yes	Yes
Christie et al. (case series 2; *37*)	Partial	Unknown	NA	Partial	Partial	Partial
Gage et al. (*31*)	Yes	Unknown	NA	Yes	Yes	Yes
Ge et al. (case report; *27*)	Yes	Yes	Yes	Yes	Yes	Yes
Ge et al. (case series; *27*)	Partial	Unknown	NA	Unknown	Partial	Partial
Kartman et al. (*33*)	Partial	Unknown	NA	No	Yes	Yes
Kartman et al. (*32*)	Partial	Unknown	NA	Unknown	Yes	Yes
Kugeler et al. (*34*)	No	Unknown	NA	Unknown	Partial	Partial
Mitchell et al. (*39*)	Yes	Unknown	NA	Unknown	Yes	Yes
Poland et al. (*35*)	Yes	Yes	Yes	Yes	Yes	Yes
Ratsitorahina et al. (*23*)	Yes	Yes	Unknown	Yes	Yes	Partial
Saeed et al. (*40*)	Yes	Yes	Yes	Yes	Yes	Yes
Sagiev et al. (*38*)	No	Unknown	NA	Unknown	Unknown	Unknown
Von Reyn et al. (*36*)	Yes	Yes	Yes	Yes	Yes	Yes
Wong et al. (*8*)	Yes	Yes	Yes	Yes	Yes	Yes
Wu et al. (*28*)	Yes	Unknown	NA	Yes	Yes	Partial
Zhang et al. (*29*)	Partial	Unknown	NA	Partial	Unknown	Partial

*ID, identification; NA, not applicable.

#### Findings

Corpses were described as the source of exposure in 3 studies comprising up to 42 cases (*23*,*38*). Axillary bubonic plague developed in 1 patient after he had conducted a postmortem examination of 2 infected corpses during the 1920s (*38*). It is unclear whether the examiner had skin lesions on the hands, was wearing PPE during the autopsy, or how soon the autopsies were conducted after death. The second study described 9 persons who contracted pneumonic plague after attending the funeral of someone who died of plague (*23*). Eight of these contacts had lodged at the house of the deceased person for 2 days after the patient’s death and might have had contact with the deceased person’s wife and son, who also died of plague shortly after. Although the authors concluded that “infection resulted from active participation in the funeral ceremonies and attendance on patients,” it is difficult to distinguish between human-to-human and corpse-to-human transmission in this scenario (*23*). The third study reported 32 persons infected by contact with plague patients or corpses; this study provided no disaggregated data nor further details on the route of transmission (*29*).

The remaining 13 studies reported 208 cases of plague transmitted by carcasses of camels, goats, cats, a bobcat, a fox, a coyote, a mountain lion, Tibetan sheep, marmots, dogs, rabbits, squirrels, and other rodents. Most exposures consisted of carcass-related activities, such as killing the animal, skinning the carcass, or conducting a necropsy, all of which require relatively long and close exposure to the infection source.

Only 1 study directly specified the duration of time between the death of the infected animal and exposure, a period of ≈35 hours (*8*). Three studies described a total of 11 cases in which exposure occurred <24 hours after the death of the infected animal (*23*,*34*,*39*). In addition, 3 other studies described 26 patients who had killed the infected animal, implying immediate exposure (*32*,*33*,*36*).

Of the patients who had bubonic plague, 5 had open skin lesions on their hands or arms while they handled the carcass with bare hands (*33*,*34*,*35*,*39*). Other persons who had no skin lesions were exposed to the same infection source but were not infected (*34*,*35*). Most cases of bubonic plague were axillary, consistent with the inoculation of *Y. pestis* through cuts in the hands or arms. Two studies attributed transmission of primary pneumonic plague to inhalation of aerosols generated by handling the carcass, including 1 study that theorized aerosol inhalation during necropsy (*8*,*27*).

#### Summary

Limited evidence exists for plague transmission from human corpses. Ten studies reported plague transmission through direct skin contact with blood from animal carcasses, leading to 121 cases of bubonic plague. Persons who had cuts or skin abrasions had an increased risk of contracting plague. The potential infectiousness of other body fluids remains unknown. It is possible that pneumonic plague might be spread by actions that cause aerosolization of infected body fluids, but this process would require considerable manipulation of the corpse or carcass.

### Infectiousness of Body Fluids of Corpses or Carcasses

We identified 2 studies that detailed the infectious period of plague-infected animal carcasses; however, we could not find any studies documenting the duration of infectiousness of human corpses. One experimental study from Madagascar published in 1965 isolated *Y. pestis* from rodents that died of septicemic plague and were buried in laterite alone or in laterite enriched with manure to simulate local conditions (*40*). *Y. pestis* was successfully isolated after 5 and 10 days, but not 15 days, after the death and burial of the rodents. Another study reported the case of a wildlife biologist who was in contact with a mountain lion carcass ≈35 hours after the animal had died (*8*). The time of death was identified from a mortality signal transmitted from the animal’s radio-collar after recording no movement for 6 hours. *Y. pestis* was isolated by culture of the animal’s tissues and subtyped by pulsed-field gel electrophoresis. The same strain was later isolated from the biologist, indicating that the mountain lion was the source of the biologist’s infection. We judged both studies to be at low risk for bias.

In summary, we do not know how long *Y. pestis* can survive in the body fluids of persons that die of plague, and thus we do not know how long the human corpse might be contagious. Because 1 study documented transmission from an animal 35 hours after death, we surmise the risk for infection from animal carcasses period might extend beyond 24 hours (*8*).

## Discussion

Historical narratives of plague outbreaks suggest that human-to-human transmission is common for pneumonic plague, but more modern researchers have contested this claim (*41*). Kool (*42*) summarized data from historical records and contemporary experiences and used qualitative analysis to conclude that “pneumonic plague is not easily transmitted from one person to another.” Some analysts have estimated transmission potential of plague using mathematical models based on historical data (*43*,*44*). The studies in this review, which examine mostly modern plague outbreaks (many earlier reports did not provide sufficient detail to meet our inclusion criteria), provide evidence that pneumonic plague is transmissible from human to human, but only after close and prolonged exposure. Historical records that did not meet inclusion criteria also provided useful information on the transmissibility of pneumonic plague. For example, some excluded studies demonstrated the isolation of *Y. pestis* from sputum of patients who had pneumonic plague (*45*,*46*), suggesting the potential for transmission of plague through inhalation of infected sputum.

We found that bloody sputum was clearly reported as the source of plague transmission in several studies. In studies describing plague transmitted from corpses, the types of contaminated body fluids causing plague transmission, although presumably blood, were not clearly described. Activities reported as the cause of infection included skinning, butchering, and flaying carcasses, as well as conducting postmortem examinations, all of which result in contact with blood. However, transmission could potentially occur through other body fluids, such as urine, feces, gastric content, or bubo pus.

We did not find evidence that plague can be transmitted by body fluids other than sputum and blood. In addition, the length of time that *Y. pestis* can survive in body fluids or that the corpse is contagious is unknown. We found only 1 study describing plague transmission from an animal that had been dead for ≈35 hours before patient exposure.

The studies in this review described 2 main routes of transmission. The first is the inhalation of particles, which can result in pneumonic plague. Plague patients generate contaminated droplets by coughing, which is associated with bloody sputum. Corpses do not produce contaminated droplets by cough, but handling the corpse in preparation for autopsy or funeral can generate contaminated droplets of body fluids, mainly blood. Regardless, a close and prolonged exposure is probably needed for disease transmission.

The second route of transmission is through the handling of corpses, such as prolonged exposure during invasive procedures. Some studies documented skin cuts or abrasions on the hands of the persons who became infected, although other studies have not commented on the presence of open wounds. Thus, it is difficult to know whether transmission through intact skin can occur, although such transmission seems improbable. We did not find any study describing plague acquired through contact with mucosa.

In some cases, we could not distinguish between transmission routes from corpses, such as whether transmission occurred through body fluids, clothing contaminated with body fluids, or fleas on the body or clothing from the corpse. Our examination of documented plague transmission from the body fluids of living plague patients found that all such reports were of primary pneumonic plague, suggesting the inhalation of particles as the transmission route. Our examination of the infectiousness of body fluids of corpses and carcasses showed that it is difficult to totally exclude the possibility that some cases of bubonic plague were transmitted by fleas. Although most patients were infected by animals (thus excluding the possibility of fleas carried on clothes), the corpses themselves might have had fleas. However, our inclusion criteria limited the likelihood of fleaborne transmission, and we appraised the plausibility of the proposed transmission route for each study. We excluded studies associated with fleas or unknown sources of transmission (*30*). We noted instances when studies reported an absence of flea bites (*33*) or when fleaborne transmission might not have been fully excluded (*19*).

**Figure 4:**
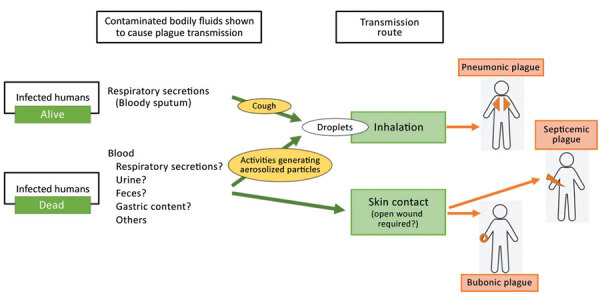
Summary of the transmission routes described in study on plague transmission from human corpses.

In summary, we provide evidence for plague transmission from human corpses (Figure 4). Inhalation of respiratory droplets produced by intense manipulation of the corpse or carcass could result in pneumonic plague, especially after close and prolonged exposure. Direct skin contact with infected body fluids (mainly blood; it is unclear whether other body fluids might also be infectious) could cause bubonic plague, or when a person has cuts on their hands, eventually septicemic plague. These findings suggest that persons handling the corpses of those who have died of plague should use PPE, including an adequate mask, gloves, and gown.

Appendix Additional information on plague transmission from corpses and carcasses.
