# Supplementary material for: Plague Transmission from Corpses and Carcasses
Source: Emerg Infect Dis. 2021 Aug;27(8):2033–41. doi: 10.3201/eid2708.200136 (PMC8314843; doi:10.3201/eid2708.200136)
Supplement: Appendix — Additional information on plague transmission from corpses and carcasses. [file 20-0136-Techapp-s1.pdf]

Article DOI: https://doi.org/10.3201/eid2708.200136

Plague Transmission from Corpses and Carcasses

Appendix

References

1. Almeida CR, Almeida AR, Vieira JB, Guida U, Butler T. Plague in Brazil during two years of bacteriological and serological surveillance. Bull World Health Organ. 1981;59:591–7.

2. Begier EM, Asiki G, Anywaine Z, Yockey B, Schriefer ME, Aleti P, et al. Pneumonic plague cluster, Uganda, 2004. Emerg Infect Dis. 2006;12:460–7.

3. Bertherat E, Thullier P, Shako JC, England K, Koné ML, Arntzen L, et al. Lessons learned about pneumonic plague diagnosis from two outbreaks, Democratic Republic of the Congo. Emerg Infect Dis. 2011;17:778–84.

4. Evans CM, Egan JR, Hall I. Pneumonic plague in Johannesburg, South Africa, 1904. Emerg Infect Dis. 2018;24:95–102.

5. Kellogg WH. An epidemic of pneumonic plague. Am J Public Health (N Y). 1920;10:599–605.

6. Kugeler KJ, Staples JE, Hinckley AF, Gage KL, Mead PS. Epidemiology of human plague in the United States, 1900–2012. Emerg Infect Dis. 2015;21:16–22.

7. Rabaan AA, Al-Ahmed SH, Alsuliman SA, Aldrazi FA, Alfouzan WA, Haque S. The rise of pneumonic plague in Madagascar: current plague outbreak breaks usual seasonal mould. J Med Microbiol. 2019;68:292–302.

8. Ramasindrazana B, Andrianaivoarimanana V, Rakotondramanga JM, Birdsell DN, Ratsitorahina M, Rajerison M. Pneumonic plague transmission, Moramanga, Madagascar, 2015. Emerg Infect Dis. 2017;23:521–4.

9. Ratsitorahina M, Chanteau S, Rahalison L, Ratsifasoamanana L, Boisier P. Epidemiological and diagnostic aspects of the outbreak of pneumonic plague in Madagascar. Lancet. 2000;355:111–3.

10. Richard V, Riehm JM, Herindrainy P, Soanandrasana R, Ratsitoharina M, Rakotomanana F, et al. Pneumonic plague outbreak, Northern Madagascar, 2011. Emerg Infect Dis. 2015;21:8–15.

11. Centers for Disease Control and Prevention. Plague—United States, 1992. MMWR Morb Mortal Wkly Rep. 1992;41:787–90.

12. Christie AB, Chen TH, Elberg SS. Plague in camels and goats: their role in human epidemics. J Infect Dis. 1980;141:724–6.

13. Gage KL, Dennis DT, Orloski KA, Ettestad P, Brown TL, Reynolds PJ, et al. Cases of cat-associated human plague in the Western US, 1977–1998. Clin Infect Dis. 2000;30:893–900.

14. Ge P, Xi J, Ding J, Jin F, Zhang H, Guo L, et al. Primary case of human pneumonic plague occurring in a Himalayan marmot natural focus area Gansu Province, China. Int J Infect Dis. 2015;33:67–70.

15. Kartman L. The role of rabbits in sylvatic plague epidemiology, with special attention to human cases in New Mexico and use of the fluorescent antibody technique for detection of *Pasteurella pestis* in field specimens. Zoonoses Res. 1960;1:1–27.

16. Kartman L. Historical and oecological observations on plague in the United States. Trop Geogr Med. 1970;22:257–75.

17. Mitchell JA, Pirie JH, Rhodes WF, Powell W. Epizootic among veld rodents in De Aar and neighbouring districts of the Northern Cape province. J Hyg (Lond). 1930;29:394–414.

18. Poland JD, Barnes AM, Herman JJ. Human bubonic plague from exposure to a naturally infected wild carnivore. Am J Epidemiol. 1973;97:332–7.

19. Saeed AA, Al-Hamdan NA, Fontaine RE. Plague from eating raw camel liver. Emerg Infect Dis. 2005;11:1456–7.

20. Sagiev Z, Meka-Mechenko T, Kunitsa T, Musagalieva R, Ismailova A, Kulbaeva M, et al. Diseases of human plague in 1974–2003 in Kazakhstan. Ekoloji. 2019;28:39–48.

21. von Reyn CF, Barnes AM, Weber NS, Quan T, Dean WJ. Bubonic plague from direct exposure to a naturally infected wild coyote. Am J Trop Med Hyg. 1976;25:626–9.

22. Wong D, Wild MA, Walburger MA, Higgins CL, Callahan M, Czarnecki LA, et al. Primary pneumonic plague contracted from a mountain lion carcass. Clin Infect Dis. 2009;49:e33–8.

23. Wu K, Wang Y, Wang M. Epidemiologic analysis on Tibetan sheep plague from 1975 to 2005 in Qinghai Province. Chinese J Endem. 2009;28:665–7.24.

24. Zhang H, Wang S, Wu D, Liang X. Dynamic analysis of human plague epidemic situation in Gansu. Chinese Journal of Endemiology. 2007;26:82–4.

Appendix Table 1. Search strategy in study on plague transmission from corpses and carcasses

Step	Search description
1	plague.mp. or *plague/
2	yersinia pestis.mp. or Yersinia pestis/
3	Step 1 or 2
4	(transmission or transmitted).ab. or (transmission or transmitted).ti.
5	disease transmission.mp. or disease transmission/
6	(contamination or contaminated or spread*).ab. or (contamination or contaminated or spread*).ti.
7	(infection or infectious).ab. or (infection or infectious).ti.
8	Step 4 or 5 or 6 or 7
9	Steps 3 and 8
10	cadaver/ or cadaver*.mp.
11	corpse*.mp. or corpse dismemberment/
12	remains.ab. or remains.ti.
13	carcass*.ab. or carcass*.ti.
14	body fluid*.ab. or body fluid*.ti.
15	posthumous care/ or burial/ or burial*.mp.
16	(cemetery or cemeteries).mp. [mp = title, abstract, heading word, drug trade name, original title, device manufacturer, drug manufacturer, device trade name, keyword, floating subheading word, candidate term word]
17	funeral*.mp.
18	entombment.mp.
19	Step 10 or 11 or 12 or 13 or 14 or 15 or 16 or 17 or 18
20	Steps 9 and 19

Note: the term “Pasteurella pestis” is indexed under “Yersinia pestis.”

Appendix Table 2. Characteristics of excluded studies in a literature review on plague transmission from human corpses and carcasses

Study	Year	Reason for exclusion
Aikimbajev et al.	2003	Transmission of plague from fleas and slaughtering; no further information on the corpses or carcasses.
Ainiwaer et al.	2011	Report of *Yersinia pestis* strain isolated from rodent carcass; no details when the rodent died; no subsequent infection of human plague.
Arbaji et al.	2005	Cases caused by consumption of raw meat from an infected camel.
Asaku et al.	2016	No report of new case of plague; rodent carcasses with no specific isolation of *Y. pestis* by culture; no details on time between death and testing.
Baltazard Bahmanyar et al.	1960	Description of interhuman transmission by parasites.
Baltazard Seydian et al.	1960	Description of interhuman transmission by parasites.
Bannerman et al.	1906	Not enough details provided.
Biggins et al.	2019	Study on maintenance of disease among rodents.
Boegler et al.	2018	Rodent carcass surveillance program; no description of direct plague transmission to humans; nor identification of *Y. pestis* from the carcasses by culture; no details on time between death and testing.
Boisier et al.	2002	No direct information on method of interhuman transmission.
Boone et al.	2009	Study of carcass removal by carnivores showed that carcasses were uninfected.
Chalmers et al.	1900	No clear data on human-to-human transmission of plague.
Cohn et al.	2018	No direct evidence on transmission by corpses.
Danforth et al.	2016	Two new cases transmitted by rodents and fleas (no direct transmission).
Davis et al.	2007	Plague transmission within burrow systems.
Didelot et al.	2017	No description of real cases of direct transmission.
Doll et al. 1912	1912	Full-text report not available.
Easterday et al.	2012	Study of genetic changes in plague bacilli.
Esposito et al.	1992	Although the paper mentions “plague has been reported in individuals who have come into contact with the infected carcasses of deer, antelope, foxes, bobcats and coyotes” no further details provided that would enable inclusion in this manuscript. Reference cited for this statement was checked.
Evans et al.	2018	Letter to the editor on transmission by respiratory droplets of parasites.
Ganière et al.	2001	Study looking at zoonoses transmitted by living cats and dogs.

Study	Year	Reason for exclusion
George et al.	1941	Description of transmission to rodents via parasites with no details on persistence or infection from rodent carcasses.
Gimlette et al.	1909	Transmission route is unclear; infection might have been brought from imported clothing with fleas; reports of rat migration and fleas.
Graf et al.	2006	No description of real cases of direct transmission.
Healing et al.	1995	Description of infection hazards of human corpses from both old interments and the recently deceased; no cases of plague transmission described from the recently deceased.
Jellison et al.	1939	Study of bird infection when eating infected rodents.
Krishnaswami et al.	1972	Prevalent rodent and flea fauna in the area; no direct evidence on transmission route was reported.
Kuznetsov et al.	2018	Cartography applied for natural foci of plague.
Lynteris et al.	2018	No direct evidence of transmission from corpses.
Madras et al.	1917	Report on veterinary services with no description of plague transmission.
Matsuo et al.	1912	No clear mode of transmission described; exposure to living infected animal.
Mayevsky et al.	1999	Description of transmission of plague among sousliks by fleas through winter.
Mitra et al.	1907	Description of a plague outbreak with no laboratory confirmation of the disease; possibility of transmission from a corpse, however, author reports: “How this man contracted plague is a mystery;” “I have heard a story;” and “there are 2 probable stories.”
Nishiura et al.	2006	Mode of transmission not described.
Njunwa et al.	1989	Study on rodents and parasites with no details on time between death and testing.
Nyirenda et al.	2017	Investigated potential risk factors associated with facilitating, maintaining, and transmitting sylvatic and murine plague to humans in the study area.
Poleykett et al.	2018	No evidence on transmission by corpses.
Reed et al.	1970	Cases attributed to fleaborne transmission.
Reynolds et al.	2011	No evidence provided on transmission of plague from animals.
Richgels et al.	2016	Simulating plague transmission through animal carcasses; no description of real cases of direct transmission.
Rollo-Koster et al.	2018	No direct evidence on transmission from corpses.
Russo et al.	1930	Study based on experimental investigation of plague in insects.
Simpson et al.	1909	Full-text manuscript could not be retrieved.
Sludsky et al.	2018	No description of any human case of plague attributed to corpses or carcasses.
Sotnikov et al.	1973	Full-text manuscript could not be retrieved.
Stepanov et al.	1990	Potential fleaborne transmission.
Strobel et al.	2004	No description of cases of plague transmission.
Strong et al.	1912	No description of human-to-human plague transmission.
Suchkdv et al.	1965	Full-text manuscript could not be retrieved.
Teh et al.	1923	No description of human-to-human plague transmission.
Titus et al.	2016	No information provided on mode of transmission.
Van Arsdel et al.	1987	Letter to the editor.
Vasin et al.	2014	No direct description of cases.
Walsh et al.	2015	No direct cases of plague transmitted by carcasses.
Webb et al.	2006	No cases of plague attributed to direct transmission by carcasses.
Werner et al.	1984	Transmission through direct contact with live cat.
Whittles et al.	2016	Cases not confirmed; incorporates direct transmission and parasitic transmission together.
Zhongliang et al.	2016	Historical narrative manuscript on a doctor involved in plague.
Zou et al.	2005	No evidence of human-to-human transmission.

Appendix Table 3. Adapted quality appraisal tool for literature review in a study on plague transmission from corpses and carcasses

For the following questions, tick answer in one of the columns.	Yes	Partial	No	Not applicable	Notes
Were patient characteristics adequately reported?					
Was there some effort to trace all contacts from the index case?					
Were the methods used for tracing contacts adequate?					
Were the laboratory methods used for defining a confirmed case of plague reliable?					
Was the route of transmission described plausible?					
Was the cause-effect of transmission plausible?					

Appendix Table 4. Studies describing infectiousness of body fluids from living persons who have plague*

Study	Pub. year	Location (date)	Index case(s), no. (type)	Infected contact(s), no. (type)	Uninfected contacts	Contagiousness	Contaminated body fluids and transmission route (according to study authors)
Almeida et al. (*1*)	1981	Brazil (1977–1979)	1 (BP)	1 (PPP)	NA	NA	NA
Begier et al. (*2*)	2006	Uganda (2004)	2 (SPP)	2 (PPP)	23 (no PEP)	Attack rate 8%	“Respiratory droplets rather than aerosols.”
Bertherat et al. (*3*)	2011	Democratic Republic of the Congo (2004–2005; 2006)	NA	292 (290 PPP, 2 SP)	NA	NA	Not directly stated; assumed transmission from sputum and “aerosolized bacteria spread through coughing”
Evans et al. (*4*)	2018	South Africa (1904)	Unable to be traced	121 (113 PP, 2 SP, 6 mixed)	NA	R_t: peak of 2–4	“There is little evidence to confirm the conventional view that such cases originated through airborne transmission from patients with bubonic plague in whom secondary pneumonic plague had developed (mixed cases).”
Kellogg et al. (*5*)	1920	USA (1919)	1 (BP, SPP)	13 (PPP)	NA	NA	“Droplet infection and personal contact.”
Kugeler et al. (*6*)	2015	USA (1900–1925)	NA	49 (PPP)	NA	NA	“Human-to-human transmission.”
Rabaan et al. (*7*)	2019	Madagascar (2017)	1 (SPP)	1,861 (PPP)†	NA	NA	“Via respiratory droplets.”
Ramasindrazana et al. (*8*)	2017	Madagascar (2015)	1 (SPP)	13 (PPP)	123 from index case (PEP in 35)	R_0: 1.44; TR: 0.41	“The matched genetic grouping between the 2 human samples is consistent with human-to-human transmission.”
Ratsitorahina et al. (*9*)	2000	Madagascar (1997)	1 (SPP)	17 (PPP)	154 (PEP)	Attack rate 8.4%	“Direct transmission of *Y. pestis* through infective cough droplets.”
Richard et al. (*10*)	2015	Madagascar (2011)	1 (PP)	19 (PPP)	41 (PEP in 39)	Attack rate 55%	Not directly stated; assumed that “pathogen is transmitted as an aerosol by droplets or by contaminated dust.”

*BP, bubonic plague; NA, not available; PEP, postexposure prophylaxis; PP, pneumonic plague; PPP, primary pneumonic plague; pub, publication; R_0, reproductive number; R_t, estimation of time varying, which is the average number of secondary infections resulting from an infectious person; SP, septicemic plague; SPP, secondary pneumonic plague; TR, transmission rate, in susceptible persons per day; *Y. pestis*, *Yersinia pestis*.
†2,417 cases of plague were registered during this outbreak, including pneumonic and bubonic forms of plague. In another manuscript describing the same outbreak, authors mentioned that pneumonic plague was confirmed in 77% of patients (*10*). This table documents the reported number of patients with pneumonic plague.

Appendix Table 5. Characteristics of Almeida et al. (*1*) analyzed in a study on plague transmission from corpses and carcasses

Characteristic	Description
Basic information	
Setting	Brazil (states of Ceara, Pernambuco, and Paraiba)
Date	Dec 1977–May 1979
Summary	This manuscript reported cases of plague in Brazil during a 2-year period. We included one case of plague attributed to human-to-human transmission. The other cases described are bubonic cases, and no other case can be ascertained to be associated with human-to-human transmission.
Index patient(s)	
No.	1
Form(s)	Bubonic plague (no further details)
Description	Whether respiratory symptoms developed in the index patient is not reported. None of the patients with confirmed plague had severe illness.
Persons infected by index patient(s)	
No.	1
Form(s)	Primary pneumonic plague
Description	Woman 33 years of age who was family contact of the index case. Absence of buboes but signs of pneumonia.
Transmission route (as described by study authors)	Not reported
Plague diagnosis	
Definition(s)	Not reported
Laboratory findings	The secondary patient had a positive sputum culture for *Yersinia pestis* and positive serologic results during convalescence.
Contacts	
No.	Not reported
Attack rate	Not reported
Method of contact tracing	Not reported
Definition of contact	Not reported
Other relevant data	None
Other relevant notes	None

Appendix Table 6. Quality appraisal of Almeida et al. (*1*) in a study on plague transmission from corpses and carcasses

Appraisal question	Judgment	Support for judgment
Were patient characteristics adequately reported?	Partial	Some data are given for the infected contact, but no information is provided on the index patient other than diagnosis of bubonic plague.
Was there some effort to trace all contacts from the index case?	Unknown	Not reported
Were the methods used for tracing contacts adequate?	Not applicable	Not applicable
Were the laboratory methods used for defining a confirmed case of plague reliable?	Yes	The diagnosis of the infected contact was confirmed by a positive sputum culture for *Yersinia pestis* and positive serologic assay on samples taken during convalescence.
Was the route of transmission plausible?	No	The index patient had bubonic plague; few other details provided to assess plausibility of route of transmission. The secondary case had primary pneumonic plague. Although we can assume that secondary pneumonic plague developed in the index patient, this diagnosis is not mentioned. Authors notes that "none of the cases with confirmed plague got severely ill."
Was the cause-effect of transmission plausible?	Unknown	Unknown route of transmission considered to judge the plausibility of the cause-effect transmission.

Appendix Table 7. Characteristics of Begier et al. (*2*) analyzed in a study on plague transmission from corpses and carcasses

Characteristic	Description
Basic information	
Setting	Uganda
Date	Dec. 2004
Summary	
Index patient(s)	
No.	2 (patients A and B)
Form	Secondary pneumonic plague (primary bubonic plague in patient A, probable bubonic plague in patient B [fever and tender lymphadenopathy without another cause of lymphadenopathy]).
Description	No social link found between index patients; no contact in the week before disease onset.<br>Both index cases had productive cough progressing to grossly bloody sputum.<br>Patients survived >1 wk without appropriate treatment; patients became severely ill and died.
Persons infected by index patient(s)	
No.	2
Form	Primary pneumonic plague
Description	The primary caregiver/mother of patient A; the primary caregiver/sister of patient B.
Transmission route (as described by study authors)	"respiratory droplets, rather than aerosols."
Plague diagnosis	
Definition(s)	Probable pneumonic plague case: respiratory illness of acute onset; accompanied by cough producing grossly bloody sputum.<br>Definite pneumonic plague case: probable case with laboratory evidence of plague infection.
Laboratory findings	Three patients had been buried already at time of outbreak identification. One patient had a PCR-positive sputum sample, negative culture results, and positive immunochromatography results.
Contacts	
No.	25
Attack rate	8%
Method of contact tracing	Prospective active surveillance in the affected region; additional retrospective pneumonic plague surveillance by interviewing private drug shop owners, business owners, traditional healers, and other area residents.
Definition of contact	Close contact: person in contact with an index patient (i.e., touched) after onset of cough producing bloody sputum and before patient death.
Other relevant data	The contacts did not receive antimicrobial prophylaxis because "more than a week had passed since the index patients' deaths when the outbreak was reported."<br>Uninfected contacts included 3 family members who slept in the same bed as infected cases the night before the death of the infected cases, including persons who slept with their heads <2 m from the coughing plague patient.<br>"In addition, around 200 people attended the 2 cases' funeral and around 75 persons touched the blanket that wrapped one index patient's body, the same blanket that was used during the patient's final days of illness. No contacts used masks, gloves, or any other form of respiratory protection."<br>"No additional pneumonic plague cases were identified during December and in the weeks after the outbreak report. However, through active surveillance we identified 3 probable bubonic plague patients who came to the subcounty's local health center in the first half of January, an increase from a baseline of 0 cases per month in the preceding 3 mo."
Other relevant notes	"Our patients' clinical course provides clues to why pneumonic plague patients usually infect few persons and why, for example, an air travel–associated outbreak would be unlikely. Our case-patients were visibly short of breath, coughing grossly bloody sputum, and barely ambulatory before transmitting the disease. Thus, when patients are substantially contagious, they are unlikely to be traveling by air and, if so, would appear ill enough to alarm nearby passengers. In most settings, persons this ill are at home or in the hospital. Recent reviews support this observation because most reported pneumonic plague transmissions involve family, friends, or medical professionals caring for ill persons at home or in the hospital."

Appendix Table 8. Quality appraisal of Begier et al. (*2*) in a study on plague transmission from corpses and carcasses

Appraisal question	Judgment	Support for judgment
Were patient characteristics adequately reported?	Yes	Form of plague and relationship between index cases and contacts are clearly reported.
Was there some effort to trace all contacts from the index case?	Yes	Contacts are well defined, traced, and described.
Were the methods used for tracing contacts adequate?	Yes	Prospective active surveillance and retrospective pneumonic plague surveillance conducted through interviews.
Were the laboratory methods used for defining a confirmed case of plague reliable?	Partial	3 cases were already buried and therefore no samples were analyzed.<br>1 case was confirmed by PCR and immunochromatographic methods.
Was the route of transmission plausible?	Yes	Inhalation of infected “respiratory droplets.”
Was the cause-effect of transmission plausible?	Yes	Both index cases are described to develop cough and “grossly bloody sputum.”

Appendix Table 9. Characteristics of Bertherat et al. (*3*) analyzed in a study on plague transmission from corpses and carcasses*

Characteristic	Description
Basic information	
Setting	Democratic Republic of the Congo: diamond mining camp in a remote area with no previous cases of plague reported in the area (2005); gold mining camp (2006).
Date	Dec. 2004–Mar 2005; Aug–Nov 2006
Summary	
Index patient(s)	
No.	Not reported
Form	Not reported
Description	Not reported
Persons infected by index patient(s)	
No.	130 (2005); 162 (2006)
Form	128 pneumonic cases, 2 septicemic cases (2005)
Description	Not reported
Transmission route (as described by study authors)	Not reported
Plague diagnosis	
Definition(s)	Definitions established by WHO†
Laboratory findings	Suspicion of plague outbreak was based on clinical evolution and outbreak characteristics. After WHO team arrival, researchers conducted microbiological testing on blood and sputum samples, immunochromatographic assays on sputum samples, and serologic assays on paired serum samples. Findings revealed 5 confirmed, 10 probable, and 115 suspected cases (2005) and 23 confirmed, 22 probable, and 117 suspected cases (2006).
Contacts	
No.	Not reported
Attack rate	Not reported
Method of contact tracing	WHO intervention (identification of around 25 close contacts for each suspected case)
Definition of contact	Not reported
Other relevant data	Close contacts received chemoprophylaxis
Other relevant notes	“Pneumonic plague is of serious concern because of the potential for human- to-human transmission from aerosolized bacteria spread through coughing. Pneumonic plague can lead to localized outbreaks, or even devastating epidemics, because the infectious dose by inhalation can be as low as 100–500 organisms.”

*WHO, World Health Organization.
†World Health Organization. International meeting on preventing and controlling plague: the old calamity still has a future. 2006 [cited 2019 Jul 30]. https://apps.who.int/iris/handle/10665/233148.

Appendix Table 10. Quality appraisal of Bertherat et al. (*3*) in a study on plague transmission from corpses and carcasses

Appraisal question	Judgment	Support for judgment
Were patient characteristics adequately reported?	No	Description of index cases and infected contacts not reported (other than number of forms of plague in infected contacts).
Was there some effort to trace all contacts from the index case?	Partial	Although the index case is not reported, contacts were traced beginning when the World Health Organization team reached the outbreak setting and put adequate measures in place.
Were the methods used for tracing contacts adequate?	Unknown	Not reported
Were the laboratory methods used for defining a confirmed case of plague reliable?	Yes	Cases were defined as confirmed, probable and suspected cases of plague according to the World Health Organization definitions.*
Was the route of transmission plausible?	Yes	Although the index case is not reported, transmission is assumed to be "human to human transmission from aerosolized bacteria spread through coughing."
Was the cause-effect of transmission plausible?	Yes	Context of pneumonic plague outbreak following the route of transmission described above.

*World Health Organization. International meeting on preventing and controlling plague: the old calamity still has a future. 2006 [cited 2019 Jul 30]. https://apps.who.int/iris/handle/10665/233148.

Appendix Table 11. Characteristics of Evans et al. (*4*) analyzed in a study on plague transmission from corpses and carcasses*

Characteristic	Description
Basic information	
Setting	Johannesburg, South Africa
Date	Jan–Mar 1904
Other notes	The study authors modeled their manuscript on the Rand Plague Committee Report† "that documented the principal findings together with the data on which their inferences were based."
Index patient(s)	
No.	Unknown
Form	Unknown
Description	The investigators were unable to trace the index case(s) and speculated that "plague infected rice was imported from Bombay during December, 1903 and January 1904. From this rice a few Indians were infected with the pneumonic form of plague."
Persons infected by index patient(s)	
No.	121
Form	113 primary pneumonic plague, 6 mixed, 2 septicemic plague; the manuscript also described 40 cases of bubonic plague attributed to a "low-key epizootic of rats."
Description	Mainly "Indians," but also "whites," "natives," and "coloreds." Mostly men. In total, 31 persons survived bubonic plague and 2 survived pneumonic plague.<br>Before death, patients with primary pneumonic plague had "scanty but blood-stained expectorations."
Transmission route (as described by study authors)	"Investigations of the Indian community identified 16 probable transmissions involving nursing, preparing bodies for funerals, attending funerals, or close family members."<br>"…transmission seemed to follow relationship pathways involving intimate contact."<br>"There is little evidence to confirm the conventional view that such cases originated through airborne transmission from patients with bubonic plague in whom secondary pneumonic plague had developed (mixed cases) and no evidence that a person from outside Johannesburg introduced pneumonic plague into the area."
Plague diagnosis	
Definition(s)	"Pure pneumonic cases were those in which no buboes could be found, but in which there was definite bronchopneumonia. The mixed cases were those in which there was definite bronchopneumonia, as well as buboes, and the *B. Pestis* [sic] was recovered both from the foci in the lungs and from the bubo. The septicaemic cases were those without either signs of pneumonia or buboes."
Laboratory findings	"*Y. pestis* in samples of sputum or tissues from organs including the lung, spleen, and liver. Bacteria were cultured, identified morphologically, and subsequently confirmed by inoculation into rabbits and guinea pigs."
Contacts	
No.	Unknown
Attack rate	Not calculated. R_t of 2–4.
Method of contact tracing	"Inspectors were appointed to search for additional sick persons."
Definition of contact	Not reported
Other relevant data	"The decrease in estimated transmissibility coincides with the start of the isolation process on March 18, suggesting that this strategy was probably effective;"<br>"A particularly noteworthy aspect of this outbreak of primary pneumonic plague was that none of the 9 escapees from the Coolie Location transmitted the disease to the wider population; the RPCR also lists lack of transmission by many other case-patients. Nevertheless, within social networks characterized by family connections, employment, caste, and so on, the disease spread rapidly."

Characteristic	Description
Other relevant notes	"Transmission rates rapidly diminished after implementation of control measures, including isolation and safer burial practices;"<br>"As the outbreak progressed, most deaths occurred in hospitals that allowed some control of burial practices. The RCPR states that '... in the case of Hindoos and the Mohammedans [sic]. The former were allowed to bury their dead: the latter, who have certain religious functions to perform were given a room in the mortuary to perform the rite. They were warned of the dangers of handling the cadavers, and it was suggested to them that the washing should be performed with a solution of corrosive sublimate;'"<br>"…pattern in which the disease is transmitted to relatives, friends, or caregivers but not to more loosely associated contacts…;"<br>"It is well known that primary pneumonic plague rapidly incapacitates the patient, who is then incapable of reaching potential contacts within the most infectious period. Nevertheless, this study shows that relatively high rates of transmission were achieved in Johannesburg in 1904, as demonstrated by the peak values for the estimated time-varying Rt."

*RPCR, Rand Plague Committee Report; R_t, estimate of time varying; *Y. pestis*, *Yersinia pestis*.
†Report upon the outbreak of plague on the Witwatersrand March 18th to July 31st, 1904. Johannesburg (South Africa): Angus Printing and Publishing Company Ltd.; 1905.

Appendix Table 12. Quality appraisal of Evans et al. (*4*) in a study on plague transmission from corpses and carcasses

Appraisal question	Judgment	Support for judgment
Were patient characteristics adequately reported?	Yes	Although the index case could not be traced, characteristics of the infected contacts are well-reported.
Was there some effort to trace all contacts from the index case?	Unknown	Not reported
Were the methods used for tracing contacts adequate?	Not applicable	Not applicable
Were the laboratory methods used for defining a confirmed case of plague reliable?	Yes	Isolation of *Yersinia pestis* from biological samples.
Was the route of transmission plausible?	Yes	Although the index case was not traced, transmission among humans attributed to spreading mechanisms of pneumonic plague.
Was the cause-effect of transmission plausible?	Yes	Context of pneumonic plague outbreak spread by human-to-human transmission of pneumonic plague.<br>Cases of bubonic plague were also reported and attributed to flea- and rat-associated transmission.

Appendix Table 13. Characteristics of Kellogg et al. (*5*) analyzed in a study on plague transmission from corpses and carcasses

Characteristic	Description
Basic information	
Setting	Oakland, California, USA
Date	Aug–Sep 1919
Other notes	
Index patient(s)	
No.	1 (patient A)
Form	Bubonic plague with secondary pneumonic plague
Description	Man who went hunting 2 and 4 d before symptoms onset. Symptoms were fever, chest pain, and right axillary bubo. Died on day 6.
Persons infected by index patient(s)	
No.	13; 1 (patient B) by the index patient (A), 12 (C–G) in contacts of patient B; 7 (H–N) by subsequent contacts.
Form	Primary pneumonic plague
Description	9 men, 4 women. All had direct close contact, such as visiting, nursing, treating, or living with an infected person. Only 1 person survived
Transmission route (as described by study authors)	"…droplet infection and personal contact with the other victims."
Plague diagnosis	
Definition(s)	Not reported
Laboratory findings	"The first three or four of this series were thought to be influenza with pneumonia." These patients did not have microbiological diagnosis because plague was not suspected yet;<br>autopsy of case J showed "numerous bipolar staining bacilli resembling plague" in lung;<br>autopsy of case M showed bacterial identification of plague in culture from guinea pig inoculated with patient lung tissue;<br>sputum samples of case H reported with identification of pneumococci.

Characteristic	Description
Contacts	
No.	Not reported
Attack rate	Not reported
Method of contact tracing	Not reported
Definition of contact	Not reported
Other relevant data	None
Other relevant notes	None

*R_t, estimate of time varying; *Y. pestis*, *Yersinia pestis*.

Appendix Table 14. Quality appraisal of Kellogg et al. (*5*) in a study on plague transmission from corpses and carcasses

Appraisal question	Judgment	Support for judgment
Were patient characteristics adequately reported?	Yes	Description of index cases and infected contacts were adequately reported.
Was there some effort to trace all contacts from the index case?	Unknown	Not reported
Were the methods used for tracing contacts adequate?	Not applicable	Not applicable
Were the laboratory methods used for defining a confirmed case of plague reliable?	Yes	Initially, plague was not suspected. After suspicion of plague, patients were isolated and the outbreak was controlled.<br>The diagnosis was confirmed with laboratory methods in 1 case by a positive culture for *Yersinia pestis* from guinea pig inoculated with lung tissue of the infected case.<br>The autopsy of “one of the other cases of the series” included smears from lung and spleen that showed numerous typical bipolar staining organisms. Although microbiological confirmation was not performed for the other cases, the clinical and epidemiologic context together with microbiological confirmation of plague in 1 case is highly suggestive of an outbreak of pneumonic plague.
Was the route of transmission plausible?	Yes	Respiratory route
Was the cause-effect of transmission plausible?	Yes	Context of pneumonic plague outbreak driven by respiratory route, beginning with an index patient with secondary pneumonic plague.

Appendix Table 15. Characteristics of Kugeler et al. (*6*) analyzed in a study on plague transmission from corpses and carcasses

Characteristic	Description
Basic information	
Setting	USA
Date	1900–2012 (no human-to-human transmission recorded during 1926–2012)
Other notes	This manuscript summarized the cases of plague in the United States from 1900–2012 on the basis of reports by the US Public Health Service, the Centers for Disease Control and Prevention, state reports, and peer-reviewed publications. We report here the cases of plague from this manuscript associated with person-to-person transmission during 1900–1925.
Index patient(s)	
No.	Not reported
Form	Not reported
Description	Not reported
Persons infected by index patient(s)	
No.	49
Form	Pneumonic plague
Description	No disaggregated data among the 496 cases of plague during this period (1900–1925).
Transmission route (as described by study authors)	Not reported
Plague diagnosis	
Definition(s)	“…clinically compatible human illness and at least 1 of the following: 1) *Y. pestis* isolated from or detected in a clinical specimen, 2) elevated antibody titer to *Y. pestis* F1 antigen in >1 serum specimen, or 3) supportive epidemiologic and other laboratory evidence (e.g., visualization of typical *Y. pestis* morphology on a stained slide)…;”<br>“The clinical form of plague (e.g., bubonic, pneumonic, septicemic) was determined on the basis of explicit notations in the case records or from available clinical details; only the primary clinical form was considered. For example, patients who had primary bubonic plague and secondary pneumonic plague were classified as having bubonic plague.”
Laboratory findings	Not reported

Characteristic	Description
Contacts	
No.	Not reported
Attack rate	Not reported
Method of contact tracing	Not reported
Definition of contact	Not reported
Other relevant data	None
Other relevant notes	None

***Y. pestis*, *Yersinia pestis*.

Appendix Table 16. Quality appraisal of Kugeler et al. (*6*) in a study on plague transmission from corpses and carcasses

Appraisal question	Judgment	Support for judgment
Were patient characteristics adequately reported?	Partial	Description of the plague cases are well-reported for the overall cases reported in the manuscript, but with no disaggregated data for the cases of plague attributed to human-to-human transmission.
Was there some effort to trace all contacts from the index case?	Unknown	Not reported
Were the methods used for tracing contacts adequate?	Not applicable	Not applicable
Were the laboratory methods used for defining a confirmed case of plague reliable?	Yes	Clear definitions of plague are provided.
Was the route of transmission plausible?	Unknown	The route of infection is described to be “person-to-person” without providing further information.
Was the cause-effect of transmission plausible?	Unknown	The route of infection is described to be “person-to-person” without providing further information.

Appendix Table 17. Characteristics of Rabaan et al. (*7*) analyzed in a study on plague transmission from corpses and carcasses*

Characteristic	Description
Basic information	
Setting	Madagascar, in a nonendemic area and in large urban centers, including the capital city of Antananarivo
Date	Aug–Nov 2017
Other notes	
Index patient(s)	
No.	1
Form	Not fully detailed, but respiratory symptoms indicate secondary pneumonic plague.
Description	“…a 31-y-old man from Toamasina who developed malaria-like symptoms.” Respiratory symptoms developed 4 d later and the man died. Respiratory symptoms developed while traveling in a public taxi.
Persons infected by index patient(s)	
No.	31 persons infected by index case; 2,417 cases in total.
Form	Pneumonic plague
Description	Not reported
Transmission route (as described by study authors)	“…ready transmission by airborne droplets…;” “…pneumonic transmission occurs person-to-person via respiratory droplets, facilitated by the densely populated nature of the urban centers.”
Plague diagnosis	
Definition(s)	Definitions established by World Health Organization†
Laboratory findings	Not reported at an individual level. Cases classified by isolation of *Yersinia pestis*, serologic assays, immunochromatography assays, and PCR, according to World Health Organization definitions.
Contacts	
No.	Not reported
Attack rate	Not reported
Method of contact tracing	Not reported. Active surveillance conducted as previously established in Madagascar.
Definition of contact	Not reported
Other relevant data	None
Other relevant notes	“For the index case, initially there was no suspicion of plague and so his body was prepared for burial using traditional methods, without any special precautions. Funerary practices have been previously observed to coincide with plague onset in Madagascar, in particular spread of pneumonic plague;” “Pneumonic plague patients should be isolated, masks should be provided for both patients and HCWs to reduce droplet transmission, bedding, clothing, sputum and excreta should be treated with chlorinated solution, and infection prevention and control measures should be observed by HCWs;” “The current plague outbreak in Madagascar highlights the rise in importance of pneumonic plague and how its transmission from person to person can have devastating impacts in the context of overcrowded urban communities.”

*HCW, healthcare worker.
†World Health Organization. International meeting on preventing and controlling plague: the old calamity still has a future. 2006 [cited 2019 Jul 30]. https://apps.who.int/iris/handle/10665/233148.

Appendix Table 18. Quality appraisal of Rabaan et al. (*7*) in a study on plague transmission from corpses and carcasses

Appraisal question	Judgment	Support for judgment
Were patient characteristics adequately reported?	Partial	Some data are provided for the index patient, but very limited characteristics are reported regarding the infected contacts. The number of cases with pneumonic plague was found from another manuscript.*
Was there some effort to trace all contacts from the index case?	Partial	Active surveillance from Madagascar is assumed but not clearly reported.
Were the methods used for tracing contacts adequate?	Unknown	Not reported
Were the laboratory methods used for defining a confirmed case of plague reliable?	Yes	Cases were defined as confirmed, probable and suspected cases of plague according to World Health Organization definitions.†
Was the route of transmission plausible?	Yes	Authors attributed the transmission to "respiratory droplets."
Was the cause-effect of transmission plausible?	Yes	Context of pneumonic plague outbreak spread by respiratory droplets.

*World Health Organization (WHO). Plague – Madagascar. 2017 [cited 2019 Jul 30]. https://www.who.int/csr/don/27-november-2017-plague-madagascar/en

†World Health Organization. International meeting on preventing and controlling plague: the old calamity still has a future. 2006 [cited 2019 Jul 30]. https://apps.who.int/iris/handle/10665/233148

Appendix Table 19. Characteristics of Ramasindrazana et al. (*8*) analyzed in a study on plague transmission from corpses and carcasses

Characteristic	Description
Basic information	
Setting	Madagascar, in a remote area that had been free of human plague for 13 y
Date	Aug 2015
Other notes	
Index patient(s)	
No.	1 (patient A)
Form	Authors suggest that patient A had contracted bubonic plague from rodents or fleas. Secondary pneumonic plague then developed in patient A.
Description	Chest pain, fever, and cough developed in a man 22 y of age 1 week after returning home from traveling. He died and "was buried in a traditional manner with a 2-night wake, exposing the family and community to the pathogen and initiating a chain of transmission."
Persons infected by index patient(s)	
No.	11 persons (2 from immediate family, 6 from extended family, 3 from the community) infected by patient A; 2 additional cases were infected from these secondary cases.
Form	Pneumonic plague
Description	9 men and 4 women, median age of 22.5 y (range 15–80 y). All had cough, 93% had blood-stained sputum.
Transmission route (as described by study authors)	"The matched genetic grouping between the 2 human samples is consistent with human-to-human transmission."
Plague diagnosis	
Definition(s)	"…according to the international standards definitions."*
Laboratory findings	4 cases of confirmed plague (2 by culture, 2 by seroconversion), 1 presumptive, 9 suspected (no samples were collected from the 9 persons who died).
Contacts	
No.	123 by patient A
Attack rate	Not reported; reproductive number of 1.44 and transmission rate of 0.41 susceptible persons/d.
Method of contact tracing	In accordance with outbreak investigation protocol established by the Institut de Pasteur de Madagascar and the Malagasy Ministry of Health.
Definition of contact	Not reported
Other relevant data	Postexposure prophylaxis was given to the 35 contacts with positive serologic results.
Other relevant notes	"During pneumonic plague outbreaks, person-to-person transmission facilitates the spread from the initial infected person to family members and the wider community;" "Pneumonic plague is rare but persists as a threat in Madagascar, where poor healthcare systems and traditional burial practices promote these outbreaks."

*World Health Organization. International meeting on preventing and controlling plague: the old calamity still has a future. 2006 [cited 2019 Jul 30]. https://apps.who.int/iris/handle/10665/233148.

Appendix Table 20. Quality appraisal of Ramasindrazana et al. (*8*) in a study on plague transmission from corpses and carcasses

Appraisal question	Judgment	Support for judgment
Were patient characteristics adequately reported?	Yes	Description of index cases and infected contacts were adequately reported.
Was there some effort to trace all contacts from the index case?	Yes	Contacts were traced and reported.
Were the methods used for tracing contacts adequate?	Yes	Following the outbreak investigation protocol established by the Institut de Pasteur de Madagascar and the Malagasy Ministry of Health.
Were the laboratory methods used for defining a confirmed case of plague reliable?	Yes	Cases were defined according to "international standard definitions" and classified as confirmed, presumptive and suspected, with laboratory confirmation detailed.
Was the route of transmission plausible?	Yes	Although not directly stated, authors assume respiratory transmission.
Was the cause-effect of transmission plausible?	Yes	Index case with respiratory symptoms and exposed to the family and community as he was "buried in a traditional manner with a 2-night wake."

Appendix Table 21. Characteristics of Ratsitorahina et al. (*9*) analyzed in a study on plague transmission from corpses and carcasses

Characteristic	Description
Basic information	
Setting	Madagascar, in a remote village of the central highlands
Date	Oct–Nov 1997
Other notes	
Index patient(s)	
No.	1
Form	Suspected bubonic plague with secondary pneumonic plague
Description	Fever and tender axillary adenitis, then chest pain, blood-stained sputum, and cough developed in a woodcutter who died.
Persons infected by index patient(s)	
No.	17
Form	Primary pneumonic plague
Description	18 persons, 9 were male and 9 were females; patients had median age of 37 y; 2 patients were children (data includes index patient).<br>The healer who had been in direct contact with the index patient "incised the patient's epigastric region and sucked out some blood;" "…[the healer had] severe fever, dyspnea, chest pain, diarrhea, and coughing with foamed and bloody sputum."<br>All patients had been in close contact with the index patient. Patients included the healer, healer's family, another patient of the healer, and villagers who had stayed in the healer's house for the funeral ceremony, and persons who had attended the healer's funeral or nursed plague patients.
Transmission route (as described by study authors)	"The contamination between patients is due to the direct transmission of *Yersinia pestis* through infective cough droplets."
Plague diagnosis	
Definition(s)	Not reported
Laboratory findings	Samples tested by culture, immunochromatographic assays, direct F1 antigen ELISA of sputum samples, and serologic assays. No samples were collected from the first 5 patients because they were buried before plague was suspected. Only 1 patient was negative for all the tests done; it was concluded that this patient did not have plague.
Contacts	
No.	154 (not including those who get infected)
Attack rate	8.4%
Method of contact tracing	Not reported (other than national active surveillance)
Definition of contact	Not reported
Other relevant data	Postexposure chemoprophylaxis was given to the identified contacts.
Other relevant notes	"[The patients' infections] resulted from their active participation in the funeral ceremonies and attendance on patients. Patients with pneumonic plague are known to be contagious at the end-stage of the disease and the number of passages of Y pestis in human lungs seems to increase its virulence;"<br>"The risk of spreading pneumonic plague is actually not as high as may be thought. By the use of IgG anti-F1 ELISA, of which the specificity was 98.5% in Madagascar, we were able to estimate the infection rate in the contact population as 8.4%. The chance of a previous exposure to *Y pestis* is negligible since human plague has not been seen in these villages for 50 y;"<br>"Elementary hygiene measures to protect family members or health workers, such as the isolation of the patient and wearing of a mask, easily prevent contagion."

Appendix Table 22. Quality appraisal of Ratsitorahina et al. (*9*) in a study on plague transmission from corpses and carcasses

Appraisal question	Judgment	Support for judgment
Were patient characteristics adequately reported?	Yes	Description of index cases and infected contacts were adequately reported.
Was there some effort to trace all contacts from the index case?	Yes	Contacts were traced and reported.
Were the methods used for tracing contacts adequate?	Unknown	Not reported
Were the laboratory methods used for defining a confirmed case of plague reliable?	Partial	Some cases were retrospectively diagnosed with no laboratory diagnosis because the patients had already died and been buried; efforts were made to confirm plague in other patients by reliable methods.
Was the route of transmission plausible?	Yes	Inhalation of “infective cough droplets.”
Was the cause-effect of transmission plausible?	Yes	The index case presented “blood-stained sputum and cough” and the primary pneumonic plague developed in the infected contact.

Appendix Table 23. Characteristics of Richard et al. (*10*) analyzed in a study on plague transmission from corpses and carcasses

Characteristic	Description
Basic information	
Setting	Madagascar, in a Northern remote region that was supposedly free of *Yersinia pestis*
Date	2011
Other notes	
Index patient(s)	
No.	1 (patient A)
Form	Primary or secondary pneumonic plague (no bubo described)
Description	Fever, headache, and chills developed in boy 13 y of age as he returned home (a 50-km distance) after working in a copper mine. Severe chest pain, cough, and hemoptysis developed. He died 8 d after symptom onset.
Persons infected by index patient(s)	
No.	19 total: 4 patients infected by patient A; 15 patients infected by secondary cases.
Form	Pneumonic plague
Description	Close contacts, including family members and caretakers. All patients had sudden onset of fever, cough, hemoptysis, and chest pain.
Transmission route (as described by study authors)	Not directly stated, but authors mention in the introduction that in the context of an outbreak among humans, “if the pathogen is transmitted as an aerosol by droplets or by contaminated dust, primary pneumonic plague may result.”
Plague diagnosis	
Definition(s)	Definitions established by World Health Organization*
Laboratory findings	Limited samples collected because postmortem samples were not available; plague outbreak was declared after death of several persons. Subsequent culturing, serologic assays, immunochromatographic assays, and molecular analysis revealed 17 suspected cases, 3 confirmed cases, and 2 presumptive cases in contacts who had positive serologic results.
Contacts	
No.	41 (not including infected cases)
Attack rate	55%
Method of contact tracing	Not reported
Definition of contact	“…have interact with the patients…;”<br>“family contacts: persons who lived in the same household as an infected person during the outbreak” (some houses consisted of a single room).
Other relevant data	Health personnel and 39 uninfected contacts received chemoprophylaxis.<br>Some contacts were people who “had spent some time with a patient or approached a patient who died during the outbreak,” whereas others were direct family members living in the same household. One person shared the same bed with a plague patient until his death and was not infected. A total of 10 contacts ‘had attended funerals for case-patients in different villages.’”
Other relevant notes	“At this lethal stage of the disease, which lasts $\leq$3 d, patients are highly infectious;”<br>“During the latency period before hemoptysis, sputum contains hardly any infectious organisms. Simple countermeasures, such as protective facial masks, are efficient in preventing transmission by droplets. Also, turning one’s head away from or turning one’s back toward a healthy person has a major prophylactic effect;”<br>“It has been suggested that patients with bubonic plague and patients who have died of plague are not directly infectious to other humans;”<br>“This suggestion is consistent with findings in the present study because contacts [10 of them] who only attended the funerals did not show symptoms or seroconversion.”

*World Health Organization. International meeting on preventing and controlling plague: the old calamity still has a future. 2006 [cited 2019 Jul 30]. https://apps.who.int/iris/handle/10665/233148.

Appendix Table 24. Quality appraisal of Richard et al. (*10*) in a study on plague transmission from corpses and carcasses

Appraisal question	Judgment	Support for judgment
Were patient characteristics adequately reported?	Yes	Description of index cases and infected contacts were adequately reported.
Was there some effort to trace all contacts from the index case?	Yes	Contacts were traced and reported.
Were the methods used for tracing contacts adequate?	Unknown	Not reported.
Were the laboratory methods used for defining a confirmed case of plague reliable?	Yes	Cases were defined as confirmed, probable and suspected cases of plague according to World Health Organization definitions.
Was the route of transmission plausible?	Yes	Although not directly stated for these cases, authors assume that plague is generally "transmitted as an aerosol by droplets or by contaminated dust."
Was the cause-effect of transmission plausible?	Yes	Index case and infected contacts had pneumonic plague.

Appendix Table 25. Studies describing plague acquired from corpses and carcasses*

Study	Study design (Setting, date)	Infection source	No. cases (form)†	Time from animal death to exposure	Description of exposure	Transmission route (as described by study authors)
Centers for Disease Control and Prevention (*11*)	Case series (USA, 1992)	Ground squirrel (*Spermophilus beldingi*)	1 (BP)‡	Unknown	Patient skinned and consumed the animal.	Not reported
Christie et al. (*12*)	Case series (Libya, 1976)	Camel	12 (7 BP with axillary or cervical buboes)§	4 immediate, 8 unknown	Four patients slaughtered and skinned the camel, 1 distributed the meat, and 7 ate or handled the meat.	Direct handling, consumption of camel meat
		Goat	5 (form not reported)	1 immediate, 4 unknown	One patient killed and skinned the goat, 1 treated the skin, and 3 lived in the same household where skin was kept.	Direct handling
Gage et al. (*13*)	Case series (USA, 1984)	Domestic cat	1 (BP with axillary buboes)	Unknown	Patient buried the dead cat.	Direct contact with infectious body fluids of the cat carcass
Ge et al. (*14*)	Case series (China, 2000–12)	Fox, marmots, dogs	32 (25 PP, 7 BP)	Unknown	Eighteen patients flayed infected animals, 12 buried infected animals, 1 fed marmot to a dog, and 1 in contact with dog that captured an infected marmot.	Not reported
	Case report (China, 2014)	Marmot	1 (PP)	Unknown	Handled infected marmot captured by a dog	Aerosol exposure
Kartman et al. (*15*)	Case series (USA, 1908–60)	Wild rabbits (cottontail rabbits)	5 (not reported)	Unknown	Patients killed and cleaned diseased animal carcasses.	Direct handling
			4 (BP with axillary buboes)	Unknown	Two patients handled and skinned 6–8 rabbits, 2 shot and skinned 8 or 9 rabbits with bare hands "which became contaminated with blood, body fluids and bits of tissue;" "the hands of both men had been cut and abraded by mesquite thorns."	Direct handling

Study	Study design (Setting, date)	Infection source	No. cases (form)†	Time from animal death to exposure	Description of exposure	Transmission route (as described by study authors)
Kartman et al. (*16*)	Case series (USA, 1908–68)	Ground squirrel	1 (BP)	Unknown	Patient hunted the animal.	Direct handling
		Ground squirrels, rabbits, prairie dogs, kangaroo rat, pocket gophers	16 (BP)¶	Unknown	Twelve patients shot or killed infected animals, 1 handled a cadaver, 1 cut himself on a rabbit bone, 1 conducted an unsterile autopsy, 1 played with an infected carcass.	Direct handling
		Prairie dog	1 (not reported)	Unknown	Patient hunted the animal.	Direct handling
Kugeler et al. (*6*)#	Synopsis (USA, 1900–2012)	Animals	64 (58 BP)	Unknown	Patients butchered or skinned animals.	Not reported
Mitchell et al. (*17*)	Report (South Africa, 1930)	Corpses	1 (BP with axillary buboes)	Unknown	Patient had conducted postmortem examination of 2 human corpses.	Not reported
Poland et al. (*18*)	Case report (USA, 1972)	Bobcat (*Lynx rufus*)	1 (BP with epitrochlear buboes)	<24 h	Animal was shot during the day and put on the vehicle. In the evening, the patient, who had open lesions on his hands, held the animal with another student while a third student eviscerated and skinned the animal. The 2 other students had no known open lesions on their hand or arms and were not infected.	Direct contact through open wounds
Ratsitorahina et al. (*9*)	Case series Madagascar, 1997	Corpses	9 (PP)	<24–36 h	Eight patients stayed for 2 d at the home of a healer who had died of plague and his ill wife and son. Therefore, patients were exposed to a corpse but also to live humans with the disease. One man who attended the healer's funeral was also exposed (possible human-to-human transmission). "Other villagers became infected during the funeral ceremonies."	'Infection resulted from active participation in the funeral ceremonies and attendance on patients.'
Saeed et al. (*19*)	Case series (Saudi Arabia, 1994)	Camel	1 (BP with axillary buboes)**	<24 h	Patient cut his arm while slaughtering and killing the animal.	Not reported (probably direct contact through open wounds)
Sagiev et al. (*20*)	Case series (Kazakhstan, 1974–2003)	Camel, hare, saiga	12 (not reported)	Unknown	Eight patients slaughtered camel, 2 cut hare carcass, 1 handled hare carcass, 1 cut carcass of a sick saiga.	Not reported
Von Reyn et al. (*21*)	Case report (USA, 1975)	Coyote	1 (BP with axillary buboes)	Unknown	Patient skinned the animal and carried the pelt. He had a forearm laceration and nailbeds exposed during the skinning.	Direct contact through open wounds
Wong et al. (*22*)	Case report (USA, 2007)	Mountain lion	1 (PP)	≈35 h	Patient carried the carcass for ≈1 km to his vehicle, then into his garage. Patient conducted necropsy with bare hands. The necropsy included the opening of the animal's thoracic cavity, which was filled with blood, and transection of the vertebral column. Necropsy lasted ≈2.5 h.	Inhalation of aerosols generated while handling the infected animal
Wu et al. (*23*)	Case series	Tibetan sheep	25 (9 BP, 6 PP, 3 septicemic plague, 2	Unknown	Patients flayed, ate, or handled animal carcasses.	Not reported

Study	Study design (Setting, date)	Infection source	No. cases (form)†	Time from animal death to exposure	Description of exposure	Transmission route (as described by study authors)
	(China, 1975–2007)		intestinal plague)			
Zhang et al. (*24*)	Case series (China, 1958–2005)	Marmot and cat carcasses and human corpses	56 (28 PP, 21 BP, 5 septicemic plague††)	Unknown	Twenty-four patients flayed, ate, or handled living or dead marmots or cats; 32 were infected by living patients or contact with corpse.	Not reported

*BP, bubonic plague; PP, pneumonic plague.
†Only primary forms described.
‡Other cases of plague are reported but associated with fleaborne or unknown transmission route.
§In addition, 3 children (children of a patient) were also reported to have plague. However, 2 were sick before the contact with the infected camel; these children received a diagnosis of typhoid fever. The remaining child might have had plague but did not have direct contact with the camel.
¶Authors reported that "specific animal contact is known to have occurred a few days before illness in 35 of the 80 bubonic cases." Of these 35 cases, this table describes 16 possibly transmitted by a dead animal and none by live animals (such as those transmitted by bites) or cases of unclear exposure source.
#This synopsis reports plague cases in the United States during 1900–2012. Exposure route was documented for 30% of the cases. This table describes cases attributed to butchering or skinning carcasses and excludes cases for which transmission from carcass was unclear (i.e., live animal handling).
**In addition, 4 patients had pharyngeal plague after eating raw meat from the infected camel. The patient described in this table had not eaten the raw camel meat.
††Among 64 total cases, including 8 that were associated with an unknown transmission route and therefore excluded from this review.

Appendix Table 26. Characteristics of study by Centers for Disease Control and Prevention (*11*) analyzed in a study on plague transmission from corpses and carcasses

Characteristic	Description
Basic information	
Setting	USA
Date	Apr 1992
Source of infection	
Species	Ground squirrel (*Spermophilus beldingi*)
Diagnostic method	Not reported
Other possible sources of infection	Not reported
Exposure	
Description	Skinned the animal carcass and consumed the meat
Duration	Not reported
Time between animal or human death and contact with patient	Not reported
Persons infected	
No.	1
Age, y/sex	16 (M)
Profession	Not reported
Signs and symptoms	Not reported
Form	Bubonic and secondary septicemic plague
Diagnostic method	Positive blood culture
Outcome	Not reported
Transmission route	
Attributed by authors	Not reported
Plausibility	Not reported
Other relevant notes	
Uninfected persons exposed to same source	Not reported

Appendix Table 27. Quality appraisal of study by Centers for Disease Control and Prevention (*11*) in a study on plague transmission from corpses and carcasses

Appraisal question	Judgment	Support for judgment
Were patient characteristics adequately reported?	Yes	Although more details could have been provided, adequate description of the infected patient.
Was there some effort to trace all contacts from the index case?	Unknown	Not reported
Were the methods used for tracing contacts adequate?	Not applicable	Not applicable
Were the laboratory methods used for defining a confirmed case of plague reliable?	Yes	Positive blood culture
Was the route of transmission plausible?	Yes	Not specifically described by the study authors, but possible transmission by direct contact during skinning of carcass, causing bubonic plague.
Was the cause-effect of transmission plausible?	Yes	Not specifically described by the study authors, but possible transmission by direct contact during skinning of carcass, causing bubonic plague.

Appendix Table 28. Characteristics of Christie et al. (*12*) analyzed in a study on plague transmission from corpses and carcasses

	Description	
Characteristic	Case series 1	Case series 2
Basic information		
Setting	Northeast Libya	Northeast Libya
Date	Feb 1976	Jun 1976
Source of infection		
Species	Camel	Goat
Diagnostic method	Not reported	Not reported
Other possible sources of infection	Not reported	Four dead rats found in the compound
Exposure		
Description	Four persons (group A) slaughtered the camel; 1 person (group B) distributed the meat; 7 persons (group C) handled or ate the camel meat	One person (group A) killed and skinned the goat; 1 person (group B) treated the skin; 3 persons (group C) no direct exposure, from the same house
Duration	Not reported	Not reported
Time between animal or human death and contact with patient	Group A: before and immediately after killing; groups B and C: not specified	Group A: immediately; groups B and C: not reported
Persons infected		
No.	12	5
Age, y/sex	Not reported	Group A consisted of an adult man; group B consisted of an adult woman; group C consisted of an adult women and 2 children.
Profession	Patient in group B was a meat dealer; professions of other patients not reported.	Not reported
Signs and symptoms	Groups A and B: not reported; group C: axillary or neck buboes	Not reported
Form	Groups A and B: not reported; group C: bubonic plague.	Not reported
Diagnostic method	Groups A and B could not undergo laboratory diagnosis; 7 persons in group C received diagnosis after undergoing hemagglutination titer	Positive serologic results in 4 persons.
Outcome	Groups A and B: death; group C: recovery	Recovery
Transmission route		
Attributed by authors	Direct handling or eating of camel meat	Direct contact

Characteristic	Description	
	Case series 1	Case series 2
Plausibility	Plausible	Not clearly reported
Other relevant notes		
Uninfected persons exposed to same source	An unknown number of villagers ate the infected camel meat.	Not reported

Appendix Table 29. Quality appraisal of Christie et al. (*12*) in a study on plague transmission from corpses and carcasses

	Case series 1		Case series 2	
Appraisal question	Judgment	Support for judgment	Judgment	Support for judgment
Were patient characteristics adequately reported?	Partial	Some patient characteristics not described	Partial	Some patient characteristics not described
Was there some effort to trace all contacts from the index case?	Unknown	No details on other contacts	Unknown	No details on other contacts
Were the methods used for tracing contacts adequate?	Not applicable	Not applicable	Not applicable	Not applicable
Were the laboratory methods used for defining a confirmed case of plague reliable?	Partial	Laboratory diagnosis made in 7 cases	Partial	Positive serologic results in 4 cases
Was the route of transmission plausible?	Yes	All infected persons had close contact with the infected animal, either by direct handling or consumption of the meat.	Partial	Direct contact in 2 cases. Unclear transmission route for the 3 persons who did not have direct contact with the infected animal lived with patients.
Was the cause-effect of transmission plausible?	Yes	All infected persons had close contact with the infected animal, either by direct handling or consumption of the meat.	Partial	Direct contact in 2 cases. Unclear transmission route for the 3 persons who did not have direct contact with the infected animal lived with patients.

Appendix Table 30. Characteristics of Gage et al. (*13*) analyzed in a study on plague transmission from corpses and carcasses

Characteristic	Description
Basic information	
Setting	California, USA
Date	Mar 1984
Source of infection	
Species	Cat
Diagnostic method	On the basis of clinical signs and symptoms
Other possible sources of infection	Inactive rodent burrows suggestive of epizootic transmission at the site
Exposure	
Description	Patient buried a dead cat
Duration	Not reported
Time between animal or human death and contact with patient	Not reported
Persons infected	
No.	1
Age, y/sex	24 (M)
Profession	Not reported
Signs and symptoms	Cellulitis, axillary bubo, thrombocytopenia, gastrointestinal bleeding, acute respiratory distress syndrome, and lactic acidosis.
Form	Bubonic plague
Diagnostic method	Positive bacterial culture
Outcome	Death
Transmission route	
Attributed by authors	Direct contact from infectious body fluids of the carcass, entry route not specified.
Plausibility	Direct contact from infectious body fluids of the cat carcass, entry route not specified.
Other relevant notes	
Uninfected persons exposed to same source	Not reported

Appendix Table 31. Quality appraisal of Gage et al. (*13*) in a study on plague transmission from corpses and carcasses

Appraisal question	Judgment	Support for judgment
Were patient characteristics adequately reported?	Yes	Relevant patient characteristics described
Was there some effort to trace all contacts from the index case?	Unknown	No information on contacts
Were the methods used for tracing contacts adequate?	Not applicable	Not applicable
Were the laboratory methods used for defining a confirmed case of plague reliable?	Yes	Diagnosed by isolation of the organism
Was the route of transmission plausible?	Yes	Direct contact with infected fluids
Was the cause-effect of transmission plausible?	Yes	Axillary bubo possibly caused by handling an infected carcass

Appendix Table 32. Characteristics of Ge et al. (*14*) analyzed in a study on plague transmission from corpses and carcasses

	Description	
Characteristic	Case report	Case series
Basic information		
Setting	China	China
Date	Jul 2014	2000–2012
Source of infection		
Species	Marmot	Fox, marmots, dog
Diagnostic method	Not assessed, but 5 dogs fed with the marmot were F1 antigen positive.	Not reported
Other possible sources of infection	Not reported	Not reported
Exposure		
Description	Handled an infected marmot that was captured by a dog	Eighteen flayed infected animals, 12 buried infected animals, 1 fed a marmot to a dog, and 1 had contact with dog that captured an infected marmot.
Duration	Short period	Not reported
Time between animal or human	Not reported, likely immediate	Not reported

Characteristic	Description: Case report	Description: Case series
death and contact with patient		
Persons infected		
No.	1	32
Age, y/sex	38 (M)	Not reported
Profession	Shepherd	Not reported
Signs and symptoms	Fever, bilateral lung signs, left pleural effusion, pericardial effusion, dilated intestines, and shock	Not reported
Form	Primary pneumonic plague	25 cases of pneumonic plague, 7 of bubonic plague
Diagnostic method	Reverse indirect hemagglutination assay for F1 antigen–positive in serum (1:40), throat (1:6400), and sputum (1:12800) samples. Positive PCR. Positive culture of sputum, throat swab, and blood samples.	Not reported
Outcome	Death	No disaggregated data
Transmission route		
Attributed by authors	Exposure to aerosols	Not reported
Plausibility	Probably primary pneumonic plague	Not reported
Other relevant notes		
Uninfected persons exposed to same source	Patient's brother dismembered the carcass and fed it to the dogs	Not reported

Appendix Table 33. Quality appraisal of Ge et al. (*14*) in a study on plague transmission from corpses and carcasses

Appraisal question	Case report: Judgment	Case report: Support for judgment	Case series: Judgment	Case series: Support for judgment
Were patient characteristics adequately reported?	Yes	All patient characteristics described in detail	Partial	Some patient characteristics not described
Was there some effort to trace all contacts from the index case?	Yes	Uninfected contacts described in detail	Unknown	Not described
Were the methods used for tracing contacts adequate?	Yes	Well-described, with paired serologic assays conducted for all contacts	Not applicable	Not applicable
Were the laboratory methods used for defining a confirmed case of plague reliable?	Yes	Several validated laboratory methods	Unknown	Not reported
Was the route of transmission plausible?	Yes	Aerosol transmission described	Partial	Transmission route not described. Most pneumonic plague patients might have been exposed to aerosols generated while handling the infected carcasses.
Was the cause-effect of transmission plausible?	Yes	Primary pneumonic plague spread by aerosol transmission	Partial	Transmission route not described. Most pneumonic plague patients might have been exposed to aerosols generated while handling the infected carcasses.

Appendix Table 34. Characteristics of Kartman et al. (*15*) analyzed in a study on plague transmission from corpses and carcasses

Characteristic	Description
Basic information	
Setting	California and New Mexico, USA
Date	1908–1960
Source of infection	
Species	Wild rabbits (cottontail rabbits)
Diagnostic method	Investigation of 1 case noted that dead domestic and wild mammals in the area were collected and tested for plague. All 4 animals found dead were infected with plague, including 2 cottontail rabbits.
Other possible sources of infection	Two patients had no evidence of flea bites; investigation of wild animals and fleas in the area identified plague-infected animals.
Exposure	
Description	For 5 patients, "the California infections were acquired after the victims killed and cleaned brush rabbits."<br>For 1 patient, "the victim became ill 3 days after he had skinned 6 cottontail rabbits shot near Maljamar."<br>For 1 patient, "the patient shot and dressed 8 cottontail rabbits and became ill with plague 4 days after."<br>For 2 patients, "they had hunted rabbits" and were hospitalized 4 and 6 d after. These patients had shot and skinned one rabbit on the spot the first night, and 8 or 9 rabbits the following day, which were skinned and dressed at home. The authors report that "they [the cadavers] were dressed with bare hands which became contaminated with blood, body fluids, and bits of tissue. The evidence showed that the hands of both men had been cut and abraded by mesquite thorns, that one of them had pulled several rabbits out of burrows with his bare hands, and also had 'cleaned' his hands by rubbing them with soil."
Duration	Not reported
Time between animal or human death and contact with patient	Not reported
Persons infected	
No.	9
Age, y/sex	4 adult men; other 5 patients not described.
Profession	Not reported
Signs and symptoms	Axillary buboes in 4 cases, not reported for 5 cases.
Form	Bubonic plague in 4 cases, not reported for 5 cases
Diagnostic method	On the basis of clinical symptoms in 2 cases, not reported for 7 cases.
Outcome	1 patient died and 2 recovered; other patient outcomes were not reported.
Transmission route	
Attributed by authors	Direct handling of diseased animal carcasses
Plausibility	For 2 patients, "the victims in both cases had axillary buboes, which are consistent with their histories of having handled and skinned wild rabbits."<br>For 2 patients, "the location of lymphadenopathy and the incubation period were consistent with entrance of the etiologic agent by manual contact with infected rabbits."
Other relevant notes	
Uninfected persons exposed to same source	Not reported

Appendix Table 35. Quality appraisal of Kartman et al. (*15*) in a study on plague transmission from corpses and carcasses

Appraisal question	Judgment	Support for judgment
Were patient characteristics adequately reported?	Partial	Characteristics, including form of plague, are given for 4 cases but not the other 5 cases.
Was there some effort to trace all contacts from the index case?	Unknown	No description of contacts.
Were the methods used for tracing contacts adequate?	Not applicable	Not applicable
Were the laboratory methods used for defining a confirmed case of plague reliable?	No	On the basis of clinical signs and symptoms, or no details on confirmed diagnosis. However, clear methodology on microbiological diagnosis of plague in animals is given.
Was the route of transmission plausible?	Yes	Direct handling of the infected carcasses.
Was the cause-effect of transmission plausible?	Yes	"The victims in both cases had axillary buboes, which are consistent with their histories of having handled and skinned wild rabbits" (2 cases);<br>"The location of lymphadenopathy and the incubation period were consistent with entrance of the etiologic agent by manual contact with infected rabbits" (2 cases).

Appendix Table 36. Characteristics of Kartman et al. (*16*) analyzed in a study on plague transmission from corpses and carcasses

Characteristic	Description
Basic information	
Setting	USA
Date	1908–1968
Source of infection	
Species	Ground squirrel (for group A); ground squirrels, rabbits, prairie dogs, kangaroo rat, pocket gophers (for group B); prairie dog (for group C).
Diagnostic method	Not reported
Other possible sources of infection	Not reported
Exposure	
Description	Plague developed in the patient in group A 3–4 d after hunting ground squirrels.<br>For some patients in group B, “specific animal contact is known to have occurred a few days prior to illness in 35 of the 80 bubonic cases.” 4 had shot ground squirrels (2 of whom consumed the squirrels), 5 killed rabbits for sport, 1 for food, a boy cut himself on a rabbit bone, and 1 handled a rabbit brought to the house by her dog, 2 killed prairie dogs.<br>Patients in group B also included “a biologist, studying prairie dogs, [who] became ill after performing an unsterile autopsy on a dead prairie dog;”<br>“1 child [who] played with a dead kangaroo rat;” and<br>“1 man [who] killed pocket gophers.”<br>The patient in group C “had hunted prairie dog.”
Duration	Not reported
Time between animal or human death and contact with patient	Not reported
Persons infected	
No.	18 (more cases are reported in the document, but with no specification on whether the persons the infecting animal was living or dead).
Age, y/sex	Patient A was an adult man; others not reported.
Profession	Patient A was a laborer; others not reported.
Signs and symptoms	Not reported
Form	Patient A had bubonic plague with secondary plague pneumonia; patients in group B had bubonic plague.
Diagnostic method	Not reported
Outcome	Not reported
Transmission route	
Attributed by authors	Direct handling
Plausibility	Plausible
Other relevant notes	
Uninfected persons exposed to same source	Not reported

Appendix Table 37. Quality appraisal of Kartman et al. (*16*) in a study on plague transmission from corpses and carcasses

Appraisal question	Judgment	Support for judgment
Were patient characteristics adequately reported?	Partial	Some salient characteristics of patients are not described.
Was there some effort to trace all contacts from the index case?	Unknown	No reporting on contacts.
Were the methods used for tracing contacts adequate?	Not applicable	Not applicable
Were the laboratory methods used for defining a confirmed case of plague reliable?	Unknown	Laboratory methods of diagnosing cases were not described.
Was the route of transmission plausible?	Yes	Direct contact by handling infected animals (including killing, conducting necropsies, and cutting oneself with a bone), resulting in bubonic plague.
Was the cause-effect of transmission plausible?	Yes	Direct contact by handling infected animals (including killing, conducting necropsies, and cutting oneself with a bone), resulting in bubonic plague.

Appendix Table 38. Characteristics of Kugeler et al. (*6*) analyzed in a study on plague transmission from corpses and carcasses

Characteristic	Description
Basic information	
Setting	USA
Date	1900–2012
Source of infection	
Species	Animal carcass
Diagnostic method	Not reported
Other possible sources of infection	Not reported
Exposure	
Description	Butchering or skinning of a carcass
Duration	Not reported
Time between animal or human death and contact with patient	Not reported
Persons infected	
No.	64
Age, y/sex	Not reported
Profession	Not reported
Signs and symptoms	Not reported
Form	Bubonic plague in 91% of cases
Diagnostic method	Not reported.
Outcome	Not reported
Transmission route	
Attributed by authors	Not reported
Plausibility	Not reported
Other relevant notes	
Uninfected persons exposed to same source	Not reported

Appendix Table 39. Quality appraisal of Kugeler et al. (*6*) in a study on plague transmission from corpses and carcasses

Appraisal question	Judgment	Support for judgment
Were patient characteristics adequately reported?	No	Patient characteristics not described
Was there some effort to trace all contacts from the index case?	Unknown	Cannot assess from available information
Were the methods used for tracing contacts adequate?	Not applicable	Not applicable
Were the laboratory methods used for defining a confirmed case of plague reliable?	Unknown	Not reported
Was the route of transmission plausible?	Partial	64 cases are reported and attributed to butchering or skinning an animal. No further details are provided by the authors. Most of the cases were bubonic form of plague, with possible transmission through handling of the carcass. Other forms of plague are not described; pneumonic plague can result as inhalation of infected aerosols generated by butchering or skinning the carcass.
Was the cause-effect of transmission plausible?	Partial	64 cases are reported and attributed to butchering or skinning an animal. No further details are provided by the authors. Most of the cases were bubonic form of plague, with possible transmission through handling of the animal carcass. Other forms of plague are not described; pneumonic plague can result as inhalation of infected aerosols generated by butchering or skinning the carcass. In addition, fleaborne transmission might not have been fully excluded.

Appendix Table 40. Characteristics of Mitchell et al. (*17*) analyzed in a study on plague transmission from corpses and carcasses

Characteristic	Description
Basic information	
Setting	South Africa
Date	Nov 1930
Source of infection	
Species	2 human corpses
Diagnostic method	Plague confirmed by postmortem and laboratory diagnostic test (test not specified)
Other possible sources of infection	Field survey conducted in the area; findings indicated active transmission of plague among rodents.
Exposure	
Description	Patient conducted postmortem examination of 2 persons who had died of plague.
Duration	Not reported
Time between animal or human death and contact with patient	Not reported
Persons infected	
No.	1
Age, y/sex	Adult man
Profession	District surgeon
Signs and symptoms	Axillary buboes
Form	Bubonic plague
Diagnostic method	Not reported
Outcome	Recovery
Transmission route	
Attributed by authors	Not reported
Plausibility	Plausible
Other relevant notes	
Uninfected persons exposed to same source	Not reported

Appendix Table 41. Quality appraisal of Mitchell et al. (*17*) in a study on plague transmission from corpses and carcasses

Appraisal question	Judgment	Support for judgment
Were patient characteristics adequately reported?	Yes	Adequate description
Was there some effort to trace all contacts from the index case?	Unknown	No details on other exposures.
Were the methods used for tracing contacts adequate?	Not applicable	Not applicable
Were the laboratory methods used for defining a confirmed case of plague reliable?	Unknown	No description of which laboratory tests were used.
Was the route of transmission plausible?	Yes	Axillary bubonic plague caused by handling infected bodies during the time involved for 2 autopsies is plausible.
Was the cause-effect of transmission plausible?	Yes	Axillary bubonic plague caused by handling infected bodies during the time involved for 2 autopsies is plausible.

Appendix Table 42. Characteristics of Poland et al. (*18*) analyzed in a study on plague transmission from corpses and carcasses

Characteristic	Description
Basic information	
Setting	Northern Arizona, USA
Date	Feb 1972
Source of infection	
Species	Bobcat (*Lynx rufus*)
Diagnostic method	*Yersinia pestis* isolated from bobcat brain tissue and bone marrow samples taken 2 weeks after death.
Other possible sources of infection	Field survey conducted in the area; findings indicated active transmission of plague among fleas and rodents.
Exposure	
Description	Dead animal was in the same vehicle during the day (location unspecified). The patient and another student held the animal while a third student eviscerated and skinned the animal.<br>The student who skinned the animal “became extensively contaminated with blood and tissue contents from the animal.” The other 2 students (including the patient) “were also contaminated but to a considerably lesser degree, since their primary task was to hold the animal [for the third student].” “Following the skinning, the first student washed with soap and water; it was not ascertained how thoroughly the other 2 students (including the plague case) washed.”
Duration	Several hours in the car; time spent skinning the animal.
Time between animal or human death and contact with patient	Same day
Persons infected	
No.	1
Age, y/sex	19 (M)
Profession	Student
Signs and symptoms	At symptom onset: generalized myalgia, headache, pain in right elbow and shoulder, fever, and upper respiratory symptoms;<br>at admission: fever, anorexia, nausea, diarrhea;<br>during admission: chills, fever, anxiety, continued severe pain in right arm and shoulder, and epitrochlear and axillary lymphadenopathy.
Form	Bubonic (epitrochlear) plague
Diagnostic method	*Y. pestis* identified from aspirate of the patient’s right epitrochlear lymph node. Paired serum samples taken 26 d apart indicated a rise in titer for anti-F1 antibodies against *Y. pestis* from 1:4 to 1:32 by passive hemagglutination.
Outcome	Recovery
Transmission route	
Attributed by authors	Direct contact with contaminated animal through breaks in the skin.
Plausibility	The 2 exposed but infected students “had no known open lesions on their hands or arms.” The infected student, however, “complained of ‘hang- nails,’ was a ‘nail chewer,’ and reported having numerous raw areas around his fingernails.”
Other relevant notes	
Uninfected persons exposed to same source	Two other exposed students were not infected; 2 additional persons with them during the day, but not during skinning, were not infected.

Appendix Table 43. Quality appraisal of Poland et al. (*18*) in a study on plague transmission from corpses and carcasses

Appraisal question	Judgment	Support for judgment
Were patient characteristics adequately reported?	Yes	All patient characteristics described in detail.
Was there some effort to trace all contacts from the index case?	Yes	Two uninfected contacts were described in detail.
Were the methods used for tracing contacts adequate?	Yes	Data provided for contacts present during and after the death of the infected animal.
Were the laboratory methods used for defining a confirmed case of plague reliable?	Yes	Diagnosed by isolation of the organism.
Was the route of transmission plausible?	Yes	Direct contact with breaks in skin.
Was the cause-effect of transmission plausible?	Yes	Presence of epitrochlear nodes indicative of bubonic plague spread by direct contact through the hands.

Appendix Table 44. Characteristics of Ratsitorahina et al. (*9*) analyzed in a study on plague transmission from corpses and carcasses

Characteristic	Description
Basic information	
Setting	Madagascar
Date	Oct 1997
Source of infection	
Species	Human
Diagnostic method	On the basis of clinical signs and symptoms
Other possible sources of infection	Exposure to live plague patients
Exposure	
Description	Eight persons stayed in infected household for 2 d for funeral ceremony of a healer who died of plague. Plague also developed in an additional person who attended the healer's funeral.
Duration	2 d
Time between animal or human death and contact with patient	<24–36 h
Persons infected	
No.	9
Age, y/sex	1 child (4 y), 8 adults (17–65 y); 2 men, 7 women.
Profession	Not reported
Signs and symptoms	Pneumonic syndrome (chest pain, blood-stained sputum, cough)
Form	Pneumonic plague
Diagnostic method	Rapid diagnostic test on the basis of F1 antigen positivity
Outcome	8 patients recovered, 1 died.
Transmission route	
Attributed by authors	"Infection resulted from active participation in the funeral ceremonies and attendance on patients;" "Other villagers became infected during the funeral ceremonies."
Plausibility	Plausible
Other relevant notes	
Uninfected persons exposed to same source	54 contacts tested: none had plague, but 13 were seropositive. Total number exposed was unclear.

Appendix Table 45. Quality appraisal of Ratsitorahina et al. (*9*) in a study on plague transmission from corpses and carcasses

Appraisal question	Judgment	Support for judgment
Were patient characteristics adequately reported?	Yes	Adequate description of patient characteristics
Was there some effort to trace all contacts from the index case?	Yes	Contacts were assessed clinically and serologically
Were the methods used for tracing contacts adequate?	Unknown	Details not provided
Were the laboratory methods used for defining a confirmed case of plague reliable?	Yes	Diagnosed using F1 rapid diagnostic test of sputum samples
Was the route of transmission plausible?	Yes	Transmission of pneumonic plague by respiratory droplets
Was the cause-effect of transmission plausible?	Partial	Pneumonic plague can be transmitted by respiratory droplets. However, it is unclear whether all the cases were infected by a corpse rather than living persons. Eight persons stayed in infected household for 2 d for funeral ceremony of a healer who died of plague. This timing coincides with the last 2 d of life of the healer's wife and son, who also died of plague. Similarly, a patient who attended the healer's funeral could also have been infected by human-to-human transmission.

Appendix Table 46. Characteristics of Saeed et al. (*19*) analyzed in a study on plague transmission from corpses and carcasses

Characteristic	Description
Basic information	
Setting	Saudi Arabia
Date	Feb 1994
Source of infection	
Species	Camel
Diagnostic method	*Yersinia pestis* isolated from bone marrow of camel, the blood and liver of live jirds collected from the camel corral, and from fleas combed from these jirds.
Other possible sources of infection	None
Exposure	
Description	Patient with an open cut on his arm slaughtered the infected camel.
Duration	Not reported
Time between animal or human death and contact with patient	Same day
Persons infected	
No.	1
Age, y/sex	Adult man
Profession	Not reported
Signs and symptoms	Fever, axillary lymphadenitis, and cellulitis
Form	Bubonic plague
Diagnostic method	Tested positive by indirect hemagglutination assay
Outcome	Recovery
Transmission route	
Attributed by authors	Not clearly stated, but probably direct contact with contaminated animal through breaks in the skin.
Plausibility	Plausible
Other relevant notes	
Uninfected persons exposed to same source	Camel meat was distributed among 106 persons. In total, 37 persons ate camel meat, although only 6 ate raw meat and 2 of those did become infected. Disease caused by consumption of raw meat is not analyzed in this review.

Appendix Table 47. Quality appraisal of Saeed et al. (*19*) in a study on plague transmission from corpses and carcasses

Appraisal question	Judgment	Support for judgment
Were patient characteristics adequately reported?	Yes	Patient characteristics described.
Was there some effort to trace all contacts from the index case?	Yes	Detailed.
Were the methods used for tracing contacts adequate?	Yes	Detailed description of contact tracing.
Were the laboratory methods used for defining a confirmed case of plague reliable?	Yes	Indirect hemagglutination assay.
Was the route of transmission plausible?	Yes	Direct contact with breaks in skin.
Was the cause-effect of transmission plausible?	Yes	Axillary lymphadenitis on same arm that had open wound, suggestive of direct contact.

Appendix Table 48. Characteristics of Sagiev et al. (*20*) analyzed in a study on plague transmission from corpses and carcasses

Characteristic	Description
Basic information	
Setting	Kazakhstan
Date	1974–2003
Source of infection	
Species	Camel (for group A), hare (for groups B and C), saiga (for group D)
Diagnostic method	Plague microbes isolated from body of saiga
Other possible sources of infection	Not reported
Exposure	
Description	Group A slaughtered camel; group B cut hare carcasses; group C fed an eagle with hare carcass; group D cut a sick saiga.
Duration	Not reported
Time between animal or human death and contact with patient	Not reported
Persons infected	
No.	12 persons in total: 8 in group A; 2 in group B; 1 in group C; 1 in group D.
Age, y/sex	Patient C was 13 y of age; others not reported.
Profession	Not reported

Characteristic	Description
Signs and symptoms	Not reported
Form	Not reported
Diagnostic method	Plague microbes isolated from patient D. Not reported for other cases.
Outcome	Not reported
Transmission route	
Attributed by authors	Not reported
Plausibility	Not applicable
Other relevant notes	
Uninfected persons exposed to same source	Not reported

Appendix Table 49. Quality appraisal of Sagiev et al. (*20*) in a study on plague transmission from corpses and carcasses

Appraisal question	Judgment	Support for judgment
Were patient characteristics adequately reported?	No	Patient characteristics not described.
Was there some effort to trace all contacts from the index case?	Unknown	Not reported
Were the methods used for tracing contacts adequate?	Not applicable	Not applicable
Were the laboratory methods used for defining a confirmed case of plague reliable?	Unknown	Only 1 case has details of laboratory diagnosis.
Was the route of transmission plausible?	Unknown	Although transmission by direct handling (including cutting carcasses) is possible, no details provided on transmission route or form of plague.
Was the cause-effect of transmission plausible?	Unknown	Absence of details on type of plague is a limitation.

Appendix Table 50. Characteristics of Von Reyn et al. (*21*) analyzed in a study on plague transmission from corpses and carcasses

Characteristic	Description
Basic information	
Setting	New Mexico, USA
Date	Feb 1974
Source of infection	
Species	Coyote
Diagnostic method	Positive fluorescent antibody test of spleen and bone marrow from carcass.
Other possible sources of infection	Patient cannot recall insect bites.
Exposure	
Description	Patient skinned the animal and carried the pelt. He had a forearm laceration and nailbeds exposed during skinning. Authors raise the possibility of animal being alive but weak at the time of exposure.
Duration	Not reported
Time between animal or human death and contact with patient	Not reported
Persons infected	
No.	1
Age, y/sex	11 (M)
Profession	Not reported
Signs and symptoms	Fever, right axillary lymphadenopathy, and wound on right middle digit. Fever, neck stiffness, and lethargy later developed.
Form	Bubonic plague, plague meningitis
Diagnostic method	Lymph nodes and cerebrospinal fluid had positive culture for plague bacilli.
Outcome	Recovery
Transmission route	
Attributed by authors	Direct contact with break in skin.
Plausibility	Only the person with breach in the skin was infected.
Other relevant notes	
Uninfected persons exposed to same source	Patient's friend who joined in skinning the carcass. Several members at 2 households handled the skin. Ten exposed persons tested negative for passive plague hemagglutination.

Appendix Table 51. Quality appraisal of Von Reyn et al. (*21*) in a study on plague transmission from corpses and carcasses

Appraisal question	Judgment	Support for judgment
Were patient characteristics adequately reported?	Yes	All patient characteristics described in detail.
Was there some effort to trace all contacts from the index case?	Yes	Uninfected contacts described in detail, including serologic status.
Were the methods used for tracing contacts adequate?	Yes	Contacts who helped to skin the coyote were traced.
Were the laboratory methods used for defining a confirmed case of plague reliable?	Yes	Diagnosed by isolation of the organism.
Was the route of transmission plausible?	Yes	Direct contact with breaks in skin.
Was the cause-effect of transmission plausible?	Yes	Axillary buboes were noted on the same arm as skin break, suggestive of direct contact.

Appendix Table 52. Characteristics of Wong et al. (*22*) analyzed in a study on plague transmission from corpses and carcasses

Characteristic	Description
Basic information	
Setting	USA
Date	Nov 2007
Source of infection	
Species	Mountain lion
Diagnostic method	Samples from the liver and submandibular lymph node were PCR-positive for *Yersinia pestis*. Immunohistochemical stain identified abundant gram-negative bacilli and *Y. pestis* in subcapsular sinuses of a submandibular lymph node. Immunohistochemical stain also identified *Y. pestis* in liver and brain samples.
Other possible sources of infection	None
Exposure	
Description	"The biologist carried the carcass for approximately 1 km to his vehicle and then into his garage, where he performed a necropsy with his bare hands; there is no evidence he wore a mask or other personal protective equipment."<br>The necropsy included the opening of the animal's thoracic cavity, which was filled with blood, and transection of the vertebral column. Necropsy lasted ≈2.5 h.<br>Archived specimens of the animal by the biologist included hide, 2 paws, skinned head, and liver.
Duration	Time of transport of the carcass for 1km, then in his car.<br>Then estimated 2.5h of examination.
Time between animal or human death and contact with patient	The first evidence of contact between the patient and the dead mountain lion was 35 h after the death of the mountain lion (as evidenced by time-stamped photographs taken by the patient). Time of death was identified from a mortality signal, prompted by no movement for 6 h, transmitted from the animal's radio-collar.
Persons infected	
No.	1
Age, y/sex	37 (M)
Profession	Wildlife biologist
Signs and symptoms	Fever, chills, nausea, myalgias, cough, and blood-tinged sputum a few hours after exposure
Form	Primary pneumonic plague
Diagnostic method	"…intravascular *Y. pestis* antigens [identified] by immunohistochemistry in multiple tissue samples, including samples of the lung, liver, heart, pharynx, and brain…;"<br>"…culture of patient tissue samples (lung and liver) yielded *Y. pestis*, as confirmed by bacteriophage-lysis testing."<br>Confluent plague bacilli admixed with an acute inflammatory infiltrate in the lung; inflammation absent from other infected organs.
Outcome	Death
Transmission route	
Attributed by authors	Inhalation of aerosols generated while handling the carcass
Plausibility	"The presence of heavy intra-alveolar inflammation admixed with confluent plague bacilli—in conjunction with the complete absence of inflammation in other infected organs—provides strong evidence that the lungs were the primary site of infection and that septicemia occurred secondarily;"<br>"Other findings consistent with an aerosol exposure include the development of cough and blood-tinged sputum within hours of symptom onset, consolidation of the right lung, and the absence of buboes on clinical and postmortem examination;"<br>"Isolates of *Y. pestis* cultured from the mountain lion's tissues were subtyped by pulsed-field gel electrophoresis (PFGE) and found to be indistinguishable from isolates recovered from the biologist," further supporting the mountain lion as the source of the biologist's infection.
Other relevant notes	
Uninfected persons exposed to same source	None

Appendix Table 53. Quality appraisal of Wong et al. (*22*) in a study on plague transmission from corpses and carcasses

Appraisal question	Judgment	Support for judgment
Were patient characteristics adequately reported?	Yes	All patient characteristics described in detail.
Was there some effort to trace all contacts from the index case?	Yes	Through interviews, photographs, and cellular phone records.
Were the methods used for tracing contacts adequate?	Yes	Through interviews, photographs, and cellular phone records.
Were the laboratory methods used for defining a confirmed case of plague reliable?	Yes	Multiple methods used.
Was the route of transmission plausible?	Yes	Through aerosols
Was the cause-effect of transmission plausible?	Yes	Clinical picture strongly suggests aerosol exposure.

Appendix Table 54. Characteristics of Wu et al. (*23*) analyzed in a study on plague transmission from corpses and carcasses

Characteristic	Description
Basic information	
Setting	China
Date	1975–2007
Source of infection	
Species	Tibetan sheep
Diagnostic method	Isolated *Yersinia pestis* strains
Other possible sources of infection	Not reported
Exposure	
Description	Patients flayed, ate, or touched the carcasses of Tibetan sheep.
Duration	Not reported
Time between animal or human death and contact with patient	Not reported
Persons infected	
No.	25
Age, y/sex	9 patients <20 y of age, 8 patients 20–45 y of age, 8 patients >45 y of age; 11 men, 14 women.
Profession	Not reported
Signs and symptoms	Not reported
Form	12 primary bubonic, 6 primary pneumonic, 3 primary septicemic, 4 primary intestinal
Diagnostic method	By trade standard (WS279–2008):<br>A. suspected case: sudden high fever of unknown cause with associated symptoms + visited plague foci in past 10 d;<br>B. probable case: clinical symptoms + contact history + F1 antigen–positive by rapid diagnostic test, indirect hemagglutination assay or ELISA; or<br>C. laboratory-confirmed case: A or B + isolated *Y. pestis* strains or A + 4-fold increase of F1 antibody
Outcome	12 patients recovered, 13 died
Transmission route	
Attributed by authors	Not reported
Plausibility	Not applicable
Other relevant notes	
Uninfected persons exposed to same source	Not reported

Appendix Table 55. Quality appraisal of Wu et al. (*23*) in a study on plague transmission from corpses and carcasses

Appraisal question	Judgment	Support for judgment
Were patient characteristics adequately reported?	Yes	Basic information reported
Was there some effort to trace all contacts from the index case?	Unknown	Not reported
Were the methods used for tracing contacts adequate?	Not applicable	Not applicable
Were the laboratory methods used for defining a confirmed case of plague reliable?	Yes	Diagnosed by isolation of the organism
Was the route of transmission plausible?	Yes	Cases of bubonic and pneumonic plague can be the result of close direct contact or inhalation of infected aerosols. Cases of intestinal plague are probably the result of eating contaminated meat.
Was the cause-effect of transmission plausible?	Partial	There is not enough information given to exclude that some cases might have been caused by human-to-human transmission.

Appendix Table 56. Characteristics of Zhang et al. (*24*) analyzed in a study on plague transmission from corpses and carcasses

Characteristic	Description
Basic information	
Setting	China
Date	1958–2005
Source of infection	
Species	Plague-infected animals or humans (the original infection sources were live animals or humans)
Diagnostic method	Not reported
Other possible sources of infection	Not reported
Exposure	
Description	In total, 24 patients were infected by flaying, eating, or touching a marmot or cat.<br>An additional 32 patients were infected by contact with plague patients or human corpse.<br>Disaggregated data not reported.
Duration	Not reported
Time between animal or human death and contact with patient	Not reported
Persons infected	
No.	56 (out of 64 patients, 8 had unknown transmission routes)
Age, y/sex	1–69 (49 M, 15 F)
Profession	Not reported
Signs and symptoms	Not reported
Form	45% pneumonic plague, 33% bubonic plague, 8% septicemic plague
Diagnostic method	Criteria for laboratory-confirmed cases:<br>Strain isolated (26 patients); clinical symptoms + F1 antibody titer $\geq$1:20 (by indirect hemagglutination assay) (2 patients); or clinical symptoms + F1 antigen–positive by reverse indirect hemagglutination assay) (1 patient).<br>Clinical cases: symptoms + epidemiologic evidence (35 patients)
Outcome	Not reported
Transmission route	
Attributed by authors	Not reported
Plausibility	Not applicable
Other relevant notes	
Uninfected persons exposed to same source	Not reported

Appendix Table 57. Quality appraisal of Zhang et al. (*24*) in a study on plague transmission from corpses and carcasses

Appraisal question	Judgment	Support for judgment
Were patient characteristics adequately reported?	Partial	Inadequate information on some aspects.
Was there some effort to trace all contacts from the index case?	Unknown	Not reported
Were the methods used for tracing contacts adequate?	Not applicable	Not applicable
Were the laboratory methods used for defining a confirmed case of plague reliable?	Partial	Diagnosis was based on clinical and epidemiologic findings for 35 cases.
Was the route of transmission plausible?	Unknown	Very limited evidence for this judgment. Although plague transmission is plausible, no data reported on transmission route in association with different forms of plague.
Was the cause-effect of transmission plausible?	Partial	Not enough information provided; cannot exclude that some cases might have been caused by human-to-human transmission.
